# Epigenetic activation of meiotic recombination near *Arabidopsis thaliana* centromeres via loss of H3K9me2 and non-CG DNA methylation

**DOI:** 10.1101/gr.227116.117

**Published:** 2018-04

**Authors:** Charles J. Underwood, Kyuha Choi, Christophe Lambing, Xiaohui Zhao, Heïdi Serra, Filipe Borges, Joe Simorowski, Evan Ernst, Yannick Jacob, Ian R. Henderson, Robert A. Martienssen

**Affiliations:** 1 Howard Hughes Medical Institute, Watson School of Biological Sciences, Cold Spring Harbor Laboratory, Cold Spring Harbor, New York 11724, USA;; 2 Department of Plant Sciences, University of Cambridge, Cambridge, CB2 3EA, United Kingdom

## Abstract

Eukaryotic centromeres contain the kinetochore, which connects chromosomes to the spindle allowing segregation. During meiosis, centromeres are suppressed for inter-homolog crossover, as recombination in these regions can cause chromosome missegregation and aneuploidy. Plant centromeres are surrounded by transposon-dense pericentromeric heterochromatin that is epigenetically silenced by histone 3 lysine 9 dimethylation (H3K9me2), and DNA methylation in CG and non-CG sequence contexts. However, the role of these chromatin modifications in control of meiotic recombination in the pericentromeres is not fully understood. Here, we show that disruption of *Arabidopsis thaliana* H3K9me2 and non-CG DNA methylation pathways, for example, via mutation of the H3K9 methyltransferase genes *KYP/SUVH4 SUVH5 SUVH6*, or the CHG DNA methyltransferase gene *CMT3*, increases meiotic recombination in proximity to the centromeres. Using immunocytological detection of MLH1 foci and genotyping by sequencing of recombinant plants, we observe that H3K9me2 and non-CG DNA methylation pathway mutants show increased pericentromeric crossovers. Increased pericentromeric recombination in H3K9me2/non-CG mutants occurs in hybrid and inbred backgrounds and likely involves contributions from both the interfering and noninterfering crossover repair pathways. We also show that meiotic DNA double-strand breaks (DSBs) increase in H3K9me2/non-CG mutants within the pericentromeres, via purification and sequencing of SPO11-1-oligonucleotides. Therefore, H3K9me2 and non-CG DNA methylation exert a repressive effect on both meiotic DSB and crossover formation in plant pericentromeric heterochromatin. Our results may account for selection of enhancer trap *Dissociation* (*Ds*) transposons into the *CMT3* gene by recombination with proximal transposon launch-pads.

Eukaryotic centromeres are the sites of kinetochore attachment to spindle microtubules that allow chromosome segregation (McKinley and Cheeseman 2015). Centromere identity is governed by nucleosomes containing CENPA/CENH3-related histone variants, which occupy large arrays of tandemly repeated satellite sequences (Allshire and Karpen 2008; Malik and Henikoff 2009). A conserved feature of centromeres shared across eukaryotes is suppression of meiotic crossover (Lambie and Roeder 1988; Copenhaver et al. 1999; Vincenten et al. 2015; Nambiar and Smith 2016). Crossover suppression is important for fertility, as centromere-proximal recombination events have been associated with chromosome segregation errors and aneuploidy (Koehler et al. 1996; Lamb et al. 1996; Rockmill et al. 2006). Plants, animals, and fungi also typically possess repetitive pericentromeric heterochromatin, containing a high density of transposable elements (Allshire and Karpen 2008; Malik and Henikoff 2009; Nambiar and Smith 2016). In crop genomes, including wheat, barley, maize, and tomato, extensive pericentromeric heterochromatin occupies more than half of the chromosome and is also crossover-suppressed (Lhuissier et al. 2007; Wei et al. 2009; Mayer et al. 2012; Choulet et al. 2014). However, the genetic and epigenetic factors that shape meiotic recombination patterns in eukaryote centromeric and pericentromeric regions remain to be fully understood.

*Arabidopsis thaliana* centromeres consist of megabase tandem arrays of the 178- to 180-base pair *CEN180* satellite repeat (Copenhaver et al. 1999; Kumekawa et al. 2000; Nagaki et al. 2003; Ito et al. 2007). The centromeric regions are also densely DNA methylated and enriched for H3K9me2 and histone variant H2A.W (Lippman et al. 2004; Lister et al. 2008; Stroud et al. 2013; Yelagandula et al. 2014). Within the centromeric satellite arrays, a subset of repeats are occupied by nucleosomes containing the centromeric variant histone H3 CENH3 (Nagaki et al. 2003; Maheshwari et al. 2017). Surrounding the *CEN180* satellite arrays are repetitive, transposon-dense regions of pericentromeric heterochromatin (Lippman et al. 2004; Lister et al. 2008; Stroud et al. 2013; Yelagandula et al. 2014). Plant transposable elements are transcriptionally silenced by H3K9me2 and DNA cytosine methylation in CG and non-CG (CHG and CHH, where H = A, C, or T) sequence contexts (Lippman et al. 2004; Du et al. 2012; Stroud et al. 2013, 2014). *A. thaliana* mutants that lose maintenance of CG or non-CG DNA methylation show elevated transposon transcription and mobility at high and moderate levels, respectively (Miura et al. 2001; Singer et al. 2001; Kato et al. 2003; Mirouze et al. 2009; Reinders et al. 2009; Teixeira et al. 2009; Tsukahara et al. 2012; Marí-Ordóñez et al. 2013; Stroud et al. 2014).

In *A. thaliana*, the chromodomain cytosine methyltransferases CHROMOMETHYLASE2 (CMT2) and CHROMOMETHYLASE3 (CMT3) recognize heterochromatic H3K9me2 via BAH and chromodomains, and methylate-associated DNA in CHH and CHG contexts, respectively (Bartee et al. 2001; Lindroth et al. 2001; Du et al. 2012; Stroud et al. 2013, 2014; Zemach et al. 2013; Dubin et al. 2015). Methylation of histone H3K9 requires the SET domain methyltransferases KRYPTONITE/SUPPRESSOR OF VARIEGATION HOMOLOG4 (KYP/SUVH4), SUPPRESSOR OF VARIEGATION HOMOLOG5 (SUVH5), and SUPPRESSOR OF VARIEGATION HOMOLOG6 (SUVH6), which are recruited to methylated DNA by SRA methyl-cytosine binding domains (Jackson et al. 2002; Malagnac et al. 2002; Ebbs and Bender 2006; Johnson et al. 2007; Stroud et al. 2013, 2014; Du et al. 2014). The de novo DNA methyltransferase DOMAINS REARRANGED METHYLTRANSFERASE2 (DRM2) is also required for maintenance methylation of non-CG contexts and thus can also affect H3K9me2 (Cao and Jacobsen 2002; Cao et al. 2003; Stroud et al. 2013, 2014). The *kyp suvh5 suvh6* mutant abolishes almost all H3K9me2 and CHG/CHH methylation, but CG methylation remains intact, while *cmt3* mutants lose CHG methylation and H3K9me2 is reduced, but CHH methylation and CG methylation are largely unchanged (Bartee et al. 2001; Lindroth et al. 2001; Jackson et al. 2002; Malagnac et al. 2002; Inagaki et al. 2010; Stroud et al. 2013, 2014; Yelagandula et al. 2014). In contrast to plants, in fission yeast, which lacks DNA methylation, the SET domain histone lysine methyltransferase CRYPTIC LOCI REGULATOR4 (CLR4) is recruited to methylated H3K9me2 directly via its chromodomain (Allshire and Ekwall 2015). Thus, by separating chromodomains (CMT2 and CMT3) from SET domains (KYP/SUVH5/SUVH6), plants have introduced non-CG DNA methylation as an additional layer of epigenetic control underlying H3K9me2. Alongside these mechanisms, the METHYLTRANSERFERASE1 (MET1) cytosine methyltransferase, VARIANT IN METHYLATION1 (VIM1) family proteins, and the DECREASE IN DNA METHYLATION1 (DDM1) SWI/SNF chromatin remodeling protein are required for maintenance of CG context DNA methylation (Kankel et al. 2003; Saze et al. 2003; Lippman et al. 2004; Lister et al. 2008; Woo et al. 2008; Stroud et al. 2013). In this work, we use mutations in *A. thaliana* heterochromatic silencing pathways to investigate epigenetic control of meiotic recombination in the pericentromeric regions.

Meiotic crossovers form via inter-homolog repair of DNA double-strand breaks (DSBs) that are generated by SPO11 topoisomerase-related complexes (Szostak et al. 1983; Keeney et al. 1997; Robert et al. 2016; Vrielynck et al. 2016). Diverse eukaryotes have evidence for recombination hotspots, which are approximately kilobase-size regions with an elevated frequency of meiotic DSBs or crossovers, compared to the genome average or surrounding regions (Kauppi et al. 2004; De Massy 2013; Choi and Henderson 2015). Hotspots in different eukaryotic lineages are controlled to varying degrees by genetic and epigenetic information (Kauppi et al. 2004; De Massy 2013; Choi and Henderson 2015). At the chromosome-scale, *A. thaliana* crossover frequency is highest in gene-dense euchromatin, whereas the heterochromatic centromeres are crossover-suppressed (Copenhaver et al. 1999; Giraut et al. 2011; Salomé et al. 2012; Yelina et al. 2015). At the fine-scale, plant crossover hotspots occur at gene promoters and terminators, and recombination is promoted by euchromatic modifications, including histone variant H2A.Z (Choi et al. 2013; Hellsten et al. 2013; Wijnker et al. 2013; Shilo et al. 2015). Acquisition of DNA methylation and H3K9me2 via the RNA-directed DNA methylation (RdDM) pathway is sufficient to silence *A. thaliana* euchromatic crossover hotpots (Yelina et al. 2015). This is consistent with DNA methylation suppressing meiotic DNA double-strand breaks in mouse (Zamudio et al. 2015), and silencing crossovers in *Ascobolus* (Maloisel and Rossignol 1998). Furthermore, loss of RNAi and Clr4-dependent H3K9me2 elevates centromeric crossovers in fission yeast (Ellermeier et al. 2010), and *Drosophila* position effect variegation (PEV) suppressor mutations (*Suppressor of Variegation*) can modify centromeric crossover frequency (Westphal and Reuter 2002). Interestingly, the *A. thaliana met1* and *ddm1* CG methylation mutants associate with remodeling of meiotic recombination along chromosomes, with crossover increases in the chromosome arms and decreases across the pericentromeres (Colomé-Tatché et al. 2012; Melamed-Bessudo and Levy 2012; Mirouze et al. 2012; Yelina et al. 2012, 2015). However, how non-CG DNA methylation and other epigenetic silencing pathways contribute to recombination landscapes along plant chromosomes has not been fully explored. In this study, we address the roles of H3K9me2 and non-CG DNA methylation in suppression of meiotic DSBs and crossovers within *A. thaliana* pericentromeric heterochromatin.

## Results

### Epigenetic activation of pericentromeric crossovers in non-CG/H3K9me2 mutants

**Figure 1.**
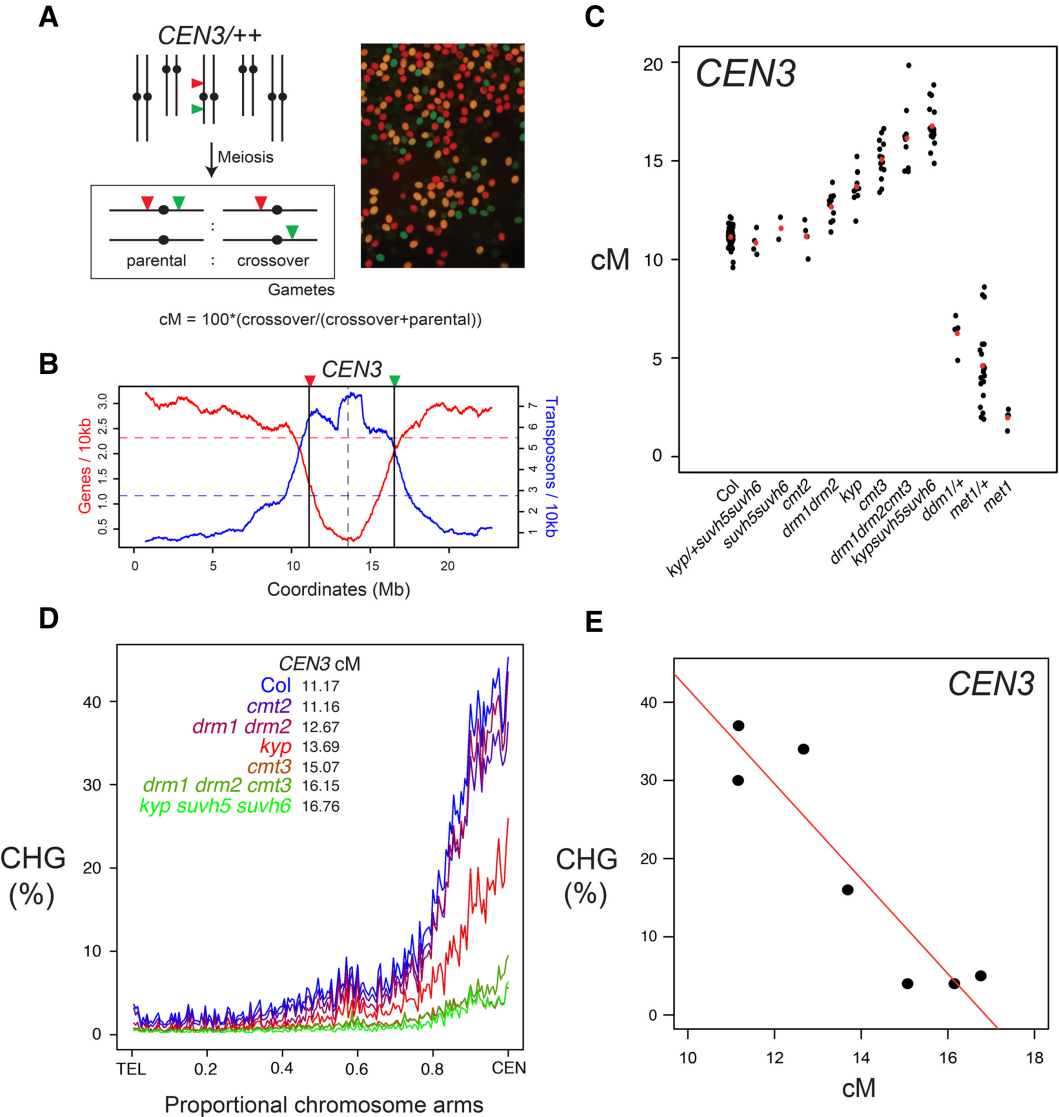
Progressive increases of pericentromeric crossover frequency in H3K9me2 and non-CG DNA methylation pathway mutants. (*A*) Measurement of crossover frequency using segregation of hemizygous fluorescent tagged line (FTL) T-DNAs. A representative fluorescent micrograph is shown of *FTL*/+ pollen, reproduced from Choi et al. (2016). (*B*) A rolling mean of gene (red) and transposon (blue) density (number of start coordinates per adjacent 10-kb window) are plotted along Chromosome 3, with the location of *CEN3* FTL T-DNAs indicated by vertical lines. Mean values are indicated by the horizontal dotted lines and the centromere assembly gap by the vertical dotted line. (*C*) *CEN3* crossover frequency (cM) in DNA methylation mutants. Data for *met1* and *met1*/+ are reproduced from Yelina et al. (2015). Black dots represent replicate measurements and red dots show mean values. (*D*) Published BS-seq data (Stroud et al. 2013) were used to analyze CHG DNA methylation density along chromosome telomere-centromere axes in wild type and H3K9me2/non-CG DNA methylation mutants. Lines are colored according to *CEN3* cM (blue = highest, red = intermediate, green = lowest). (*E*) Correlation between *CEN3* cM and CHG DNA methylation from published BS-seq data (Stroud et al. 2013).

To investigate meiotic recombination frequency in *A. thaliana* non-CG DNA methylation and H3K9me2 mutants, we used fluorescent crossover reporter lines (fluorescent tagged lines, FTLs) (Melamed-Bessudo et al. 2005; Berchowitz and Copenhaver 2008). FTLs express different colors of fluorescent protein under seed (*NapA*)- or pollen (*LAT52*)-specific promoters, from linked T-DNAs insertions (Fig. 1A). The scoring of fluorescent color inheritance in the progeny seed or pollen (male gametes) of FTL hemizygotes allows the measurement of sex-averaged or male-specific crossover frequency, respectively, in defined chromosomal intervals (Fig. 1A; Melamed-Bessudo et al. 2005; Berchowitz and Copenhaver 2008; Yelina et al. 2013).

We first analyzed crossover frequency within the 5.4-megabase (Mb) *CEN3* FTL interval, which spans the centromere and pericentromeric heterochromatin of Chromosome 3, in wild type versus non-CG/H3K9me2 pathway mutants (Fig. 1B; Yelina et al. 2015). Genetic ablation of the H3K9 methyltransferases (*kyp suvh5 suvh6*) or the non-CG DNA methyltransferases (*drm1 drm2 cmt2 cmt3*) eliminates both H3K9me2 and non-CG DNA methylation, while single and double mutants have intermediate effects (Cao et al. 2003; Stroud et al. 2013, 2014). We observed that mutations that disrupt H3K9me2 and non-CG DNA methylation to progressively greater extents resulted in progressively greater increases in *CEN3* crossover frequency (*drm1 drm2* < *kyp* < *cmt3* < *drm1 drm2 cmt3* < *kyp suvh5 suvh6*; all χ^2^ *P* < 2.0 × 10^−16^) (Fig. 1C; Supplemental Table S1; Stroud et al. 2013, 2014). The *suvh5 suvh6* and *cmt2* mutants did not show significant differences compared to wild type (Fig. 1C; Supplemental Table S1).

**Figure 2.**
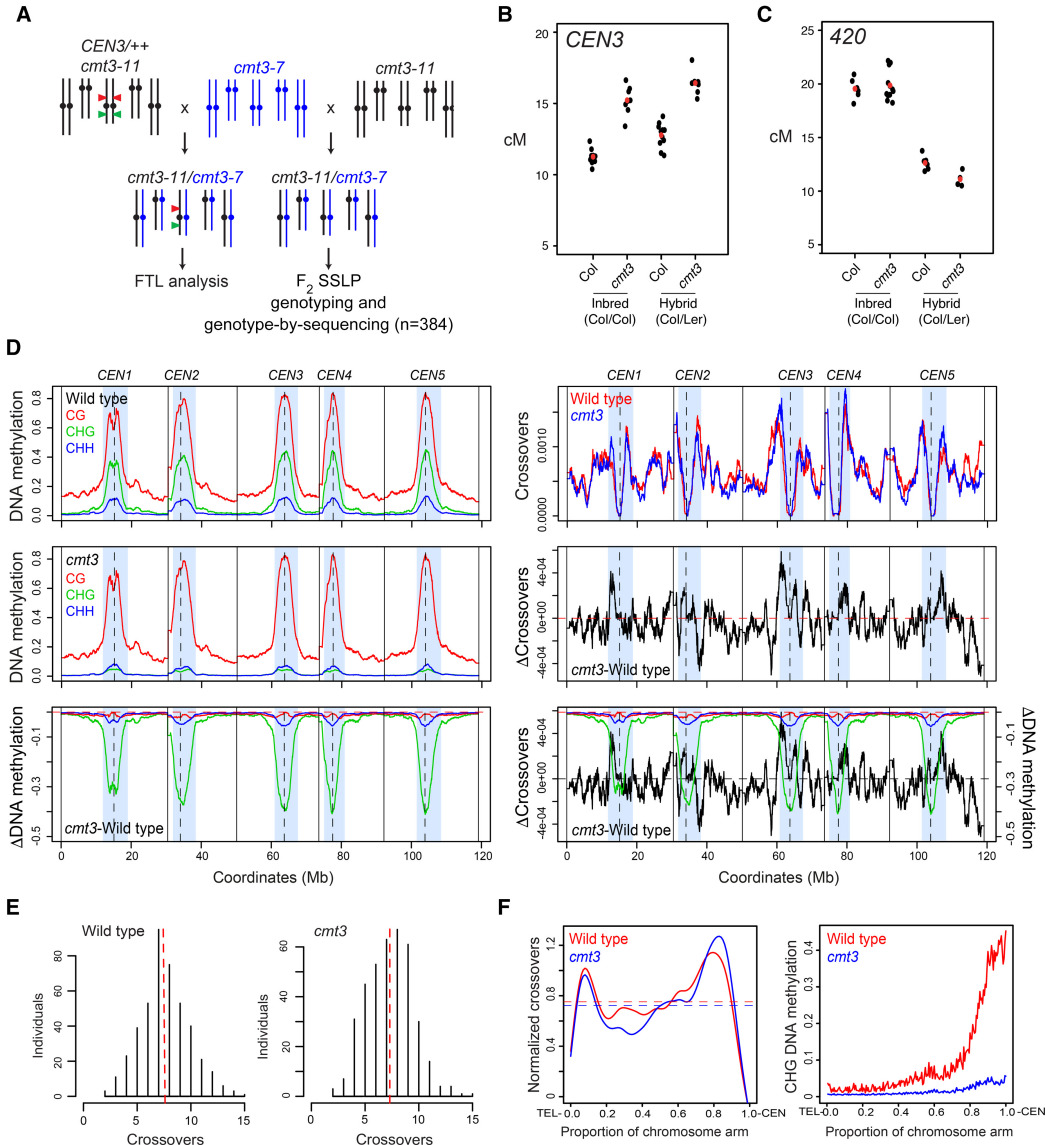
Genome-wide mapping of crossover frequency in *cmt3* non-CG mutants. (*A*) Crossing scheme used to analyze recombination in *cmt3* mutants. Col chromosomes are black and Ler chromosomes are blue. The *CEN3* FTL T-DNAs are indicated by red and green triangles. (*B*) *CEN3* crossover frequency (cM) in wild type and *cmt3*, in Col/Col inbreds, or Col/Ler F_1_ hybrids. Replicate measurements are shown in black and mean values in red. (*C*) *420* crossover frequency in wild type and *cmt3*, in Col/Col inbreds, or Col/Ler F_1_ hybrids, as shown for *B*. (*D*) Plots of the *A. thaliana* chromosomes on a continuous *x*-axis are shown. Analysis of DNA methylation frequency in CG (red), CHG (green), and CHH (blue) from published data in wild type (Col) or *cmt3-11* (Stroud et al. 2013) (*left* panels). A differential (*cmt3* − wild type = ΔDNA methylation) plot is also shown. Vertical dotted lines indicate the position of the centromere assembly gaps and vertical solid lines indicate telomeres. The pericentromeres, defined by higher than average DNA methylation, are indicated by light blue shading. The normalized frequency of crossovers mapped by GBS in wild type (red) and *cmt3* (blue) F_2_ populations is plotted (*right* panels), in addition to the *cmt3* − wild type differential (ΔCrossovers). (*E*) Histograms of crossovers per F_2_ individual for wild-type and *cmt3* populations. Red dotted lines indicate mean values. (*F*) Normalized crossover frequency analyzed along chromosome telomere (TEL) to centromere (CEN) axes in wild-type (red) and *cmt3* (blue) populations. CHG DNA methylation was analyzed and plotted similarly for wild type (red) or *cmt3* (blue) (Stroud et al. 2013).

We used published bisulfite sequencing data to analyze DNA methylation levels within the *CEN3* interval in the genotypes analyzed for crossover frequency (Stroud et al. 2013). Within this series of mutants, levels of CHG DNA methylation showed a strong negative correlation with *CEN3* genetic distance (Pearson's *r* = −0.93, *P* = 2.36 × 10^−3^), whereas CG and CHH methylation were not significantly correlated (Fig. 1D,E; Supplemental Table S2; Stroud et al. 2013). In contrast to non-CG/H3K9me2 mutants, heterozygous *ddm1*/+, *met1*/+, or homozygous *met1* mutants, which inherit chromosomes hypomethylated in the CG context, have reduced *CEN3* recombination, as reported previously (Fig. 1C; Supplemental Table S1; Colomé-Tatché et al. 2012; Melamed-Bessudo and Levy 2012; Mirouze et al. 2012; Yelina et al. 2012, 2015). We confirmed release of crossover suppression in *cmt3* across the Chromosome 5 centromere and pericentromeric heterochromatin, using additional FTLs in Col (*CTL5.11*, χ^2^ *P* = 1.30 × 10^−3^) and Ler (*LTL5.4*, χ^2^ *P* = 2.09 × 10^−9^) inbred backgrounds (Supplemental Fig. S1; Supplemental Tables S3, S4). We also crossed *cmt3* alleles in Col (*cmt3-11*) and Ler (*cmt3-7*) accessions together to generate Col/Ler F_1_ progeny that were *cmt3* mutant and carried the *CEN3* FTL (Fig. 2A; Lindroth et al. 2001). We observed significantly increased *CEN3* genetic distance in *cmt3* hybrids (χ^2^ *P* = 1.27 × 10^−86^), similar to the increase observed for inbreds (Fig. 2B; Supplemental Tables S1, S5). In contrast, recombination in the euchromatic *420* FTL interval on Chromosome 3 did not significantly change in *cmt3* inbreds and slightly decreased in hybrids (χ^2^ *P* = 1.96 × 10^−3^), compared to wild type (Fig. 2C; Supplemental Table S6). Together, these data indicate that mutations in the H3K9me2/non-CG pathway primarily activate crossover frequency in proximity to the centromeres.

### Genome-wide mapping of crossovers in *chromomethylase3* mutants

We next sought to map crossovers genome-wide in wild type compared with a H3K9me2/non-CG mutant background, using segregation of single nucleotide polymorphisms (SNPs). We used *cmt3* mutant alleles in both Col (*cmt3-11*) and Ler (*cmt3-7*) backgrounds (Bartee et al. 2001; Lindroth et al. 2001; Stroud et al. 2013, 2014) to generate wild type (Col × Ler) and *cmt3* (*cmt3-11* × *cmt3-7*) F_2_ populations of >700 individuals each (Fig. 2A). To assess centromeric recombination levels in these populations, we genotyped Col/Ler simple sequence length polymorphism (SSLP) markers on Chromosomes 1 and 3 (Supplemental Table S7). This confirmed significant increases in pericentromeric recombination in the *cmt3* population compared to wild type, consistent with our previous FTL measurements (Supplemental Table S7). To map crossovers at high resolution, we performed genotyping by sequencing (GBS) of 437 wild-type and 384 *cmt3* F_2_ individuals, which identified 3320 and 2803 crossovers, respectively (Fig. 2D–F; Supplemental Table S8; Rowan et al. 2015; Choi et al. 2016; Serra et al. 2018). The crossovers were mapped between Col/Ler SNPs to a mean resolution of 887 bp. The total number of crossovers per wild-type F_2_ individual (mean = 7.6) was comparable to that observed in similar F_2_ populations (Giraut et al. 2011; Salomé et al. 2012) and was not significantly different in *cmt3* (mean = 7.3; Mann–Whitney–Wilcoxon test, *P* = 0.101) (Fig. 2E; Supplemental Table S8).

To analyze crossover distributions throughout the genome, we defined centromeres as the crossover-suppressed regions that surround gaps in the chromosome assembly (Copenhaver et al. 1999; Kumekawa et al. 2000), the pericentromeres as the contiguous regions flanking the centromeres with higher than average DNA methylation, and chromosome arms as the remainder of the genome (Supplemental Table S9). Consistent with our FTL analysis, we observed that GBS-mapped crossovers were significantly increased in the *cmt3* pericentromeric regions (24.6% versus 27.8% of events were pericentromeric in wild type versus *cmt3*, χ^2^ *P* = 5.60 × 10^−3^), which are strongly depleted of CHG DNA methylation in *cmt3* (Fig 2D,F; Supplemental Table S10; Stroud et al. 2013). We also observed elevated centromeric crossovers (*n* = 13) in *cmt3* (χ^2^ *P* = 2.63 × 10^−4^), which were completely absent in wild type (Supplemental Table S10). The chromosome arms showed a significant decrease of crossovers in *cmt3* (χ^2^ *P* = 1.50 × 10^−3^) (Supplemental Table S10). These data confirm that crossovers increase in proximity to *cmt3* centromeres, but that the increase is strongest in the flanking pericentromeric regions (Fig. 2D,F). However, we note that some pericentromeric regions showed higher crossover frequency in wild type than *cmt3*—for example, the right pericentromere of Chromosome 2—and therefore, region-specific effects also likely play an important role, such as structural genetic variation.

### Meiotic immunocytology of chromatin and recombination in non-CG/H3K9me2 mutants

**Figure 3.**
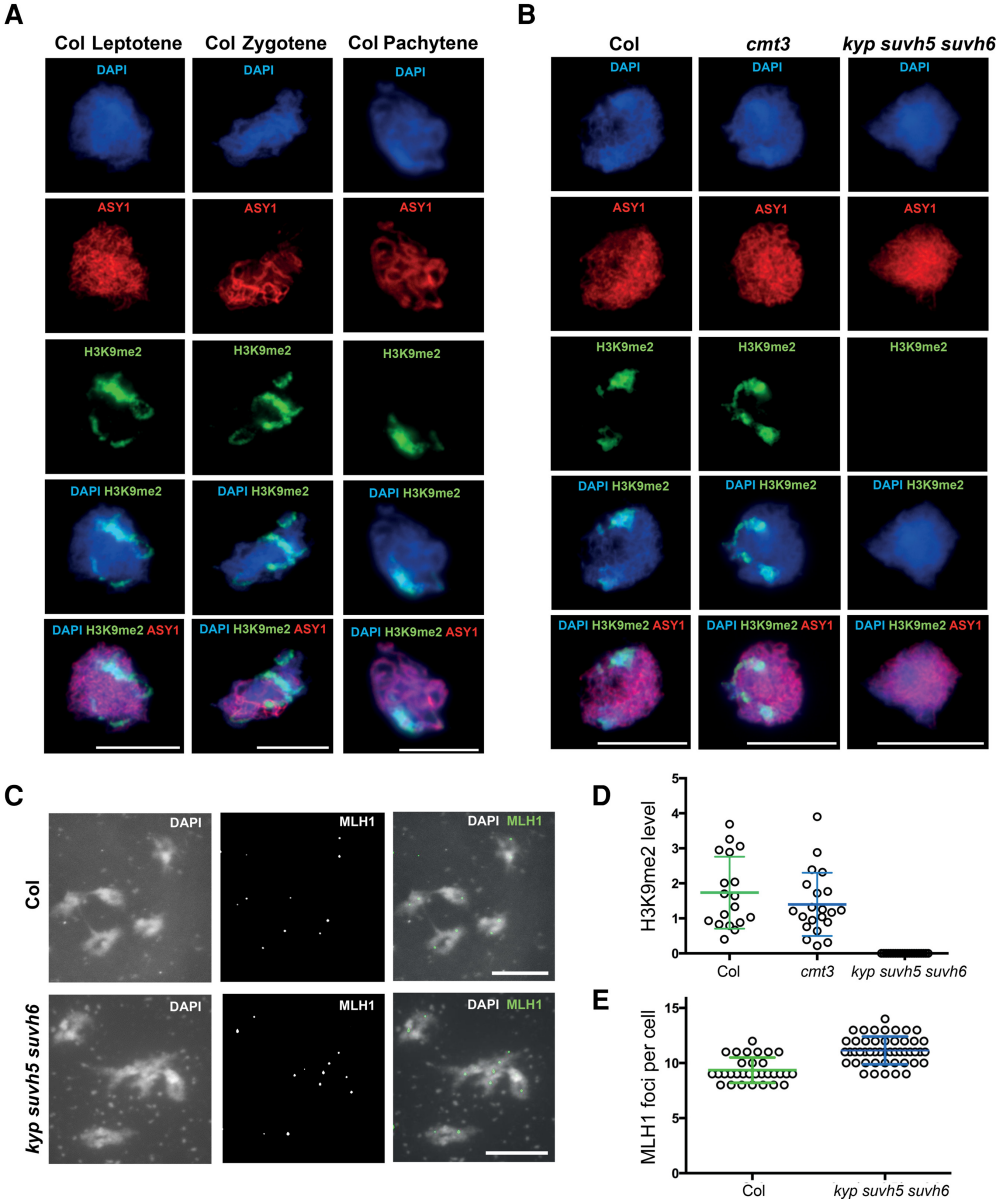
Meiotic heterochromatin is enriched for H3K9me2 and shows increased MLH1 foci in *kyp suvh5 suvh6*. (*A*) Wild-type (Col) male meiocytes immunostained for ASY1 (red) and H3K9me2 (green) and stained for DNA (DAPI, blue), at leptotene, zygotene, and pachytene stages. Scale bar, 10 µM. (*B*) As for *A*, but showing leptotene stage cells from wild type (Col), *cmt3-11*, and *kyp suvh5 suvh6*. (*C*) Male meiocytes at diakinesis stained for DAPI and immunostained for MLH1 (green) in wild type and *kyp suvh5 suvh6*. Bivalent chromosomes are evident that are associated with MLH1 foci at crossover sites. (*D*) Quantification of H3K9me2 immunostaining in wild type (Col), *cmt3*, and *kyp suvh5 suvh6* meiotic cells. (*E*) Quantification of MLH1 foci in wild type and *kyp suvh5 suvh6*.

To assess H3K9me2 patterns during meiosis, we performed immunocytological staining using antibodies against this histone modification and the chromosome axis HORMA domain protein ASYNAPTIC1 (ASY1) (Fig. 3A; Armstrong et al. 2002). During meiosis, *A. thaliana* centromeres and pericentromeres undergo progressive clustering during prophase-I (Fig. 3A; Armstrong et al. 2001). We observed that the heterochromatic clusters, detected by DAPI staining of DNA, stain strongly for H3K9me2 throughout leptotene, zygotene, and pachytene (Fig. 3A), which are the key meiotic stages during which DSB formation and crossover maturation occur (Armstrong et al. 2001; Sanchez-Moran et al. 2007). At the leptotene stage, when meiotic DSBs initiate, the H3K9me2 signal was significantly reduced to background levels in *kyp suvh5 suvh6* (Mann–Whitney–Wilcoxon test, *P* = 7.47 × 10^−9^). Although the mean H3K9me2 signal was lower in *cmt3* compared to wild-type controls, the difference was not statistically significant (Fig. 3B,D; Supplemental Fig. S2; Supplemental Tables S11, S12), consistent with intermediate reductions in H3K9me2 observed previously in *cmt3* somatic cells (Inagaki et al. 2010; Yelagandula et al. 2014). Thus, H3K9me2 accumulates strongly in *A. thaliana* heterochromatin during meiosis, and its loss or reduction results in increased pericentromeric crossover.

Crossovers can be detected by immunostaining for MLH1, which marks class I interfering crossover foci (Crismani et al. 2012). Therefore, we scored MLH1 foci associated with euchromatin or heterochromatin, based on DAPI staining, in wild type and *kyp suvh5 suvh6* mutants (Fig. 3C,E; Supplemental Table S13). We observed a slight but significant increase in MLH1 foci numbers in *kyp suvh5 suvh6* (mean = 11.1) compared to wild type (mean = 9.4) at diakinesis (Mann–Whitney–Wilcoxon test, *P* = 1.64 × 10^−7^) (Fig. 3C,E; Supplemental Table S13). Importantly, MLH1 foci were also significantly increased in *kyp suvh5 suvh6* heterochromatin (mean = 2.9), compared to wild type (mean = 1.7; Mann–Whitney–Wilcoxon test, *P* = 4.22 × 10^−5^) (Fig. 3C,E; Supplemental Table S14). This provides cytological support for our crossover mapping data and indicates that MLH1-dependent repair contributes to the increase in pericentromeric crossovers observed in H3K9me2/non-CG DNA methylation mutants.

**Figure 4.**
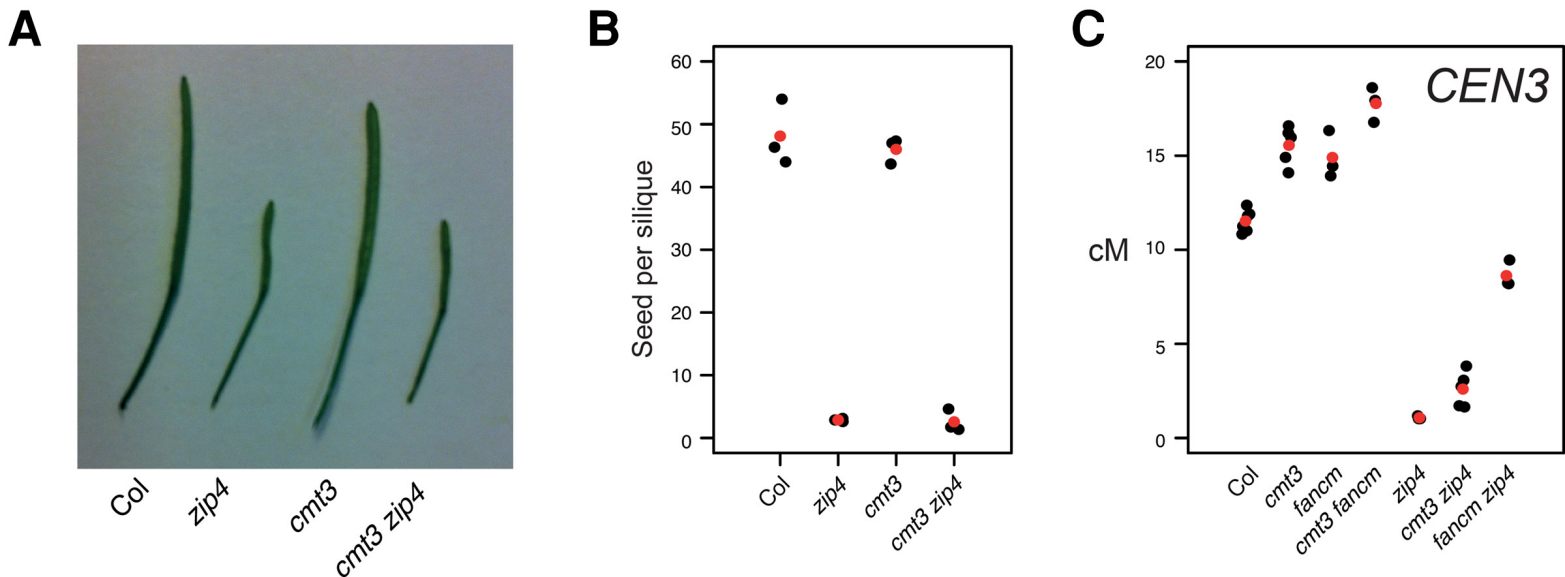
Genetic interactions between class I and class II crossover pathways and H3K9me2/non-CG methylation. (*A*) Silique length in wild type (Col), *zip4*, *cmt3*, and *cmt3 zip4*. (*B*) Seed set per silique in wild type (Col), *zip4*, *cmt3*, and *cmt3 zip4*. Replicate measurements are shown in black and mean values in red. (*C*) *CEN3* crossover frequency (cM) in wild type, *cmt3*, *zip4*, and *fancm* mutant backgrounds. Replicate measurements are shown in black and mean values in red.

As MLH1 foci were increased in *kyp suvh5 suvh6*, we further investigated the relationship of class I and class II crossover pathways to the observed recombination changes. Approximately 85% of *A. thaliana* crossovers are dependent on the class I repair pathway and are interference-sensitive (Higgins et al. 2004; Mercier et al. 2005). Mutants in the class I pathway, for example, *zip4*, cause a strong reduction in crossovers and fertility (Mercier et al. 2005; Chelysheva et al. 2007). The *fancm* mutation restores fertility in *fancm zip4* double mutants by increasing class II noninterfering crossovers (Crismani et al. 2012). Therefore, we constructed *cmt3 zip4* and *cmt3 fancm* double mutants and compared *CEN3* crossover frequency and fertility to wild type and single mutants (Fig. 4A–C; Supplemental Tables S14, S15). Unlike *fancm, cmt3* was unable to suppress *zip4* infertility (Fig. 4A,B; Supplemental Table S15). However, a small but significant *CEN3* crossover increase was observed in *cmt3 zip4*, compared with *zip4* alone (χ^2^ *P* = 2.99 × 10^−9^), which indicates that the class II pathway may contribute to increased crossovers in *cmt3* mutant centromeres (Fig. 4C; Supplemental Table S14). Additionally, the *cmt3 fancm* double mutant shows an additive increase in *CEN3* crossover frequency, compared with *cmt3* (χ^2^ *P* = 3.34 × 10^−10^) and *fancm* (χ^2^ *P* = 2.66 × 10^−7^) single mutants (Fig. 4C; Supplemental Table S14). From these data, we conclude that increased pericentromeric crossovers in H3K9me2 and non-CG DNA methylation mutants may involve contributions from both interfering and noninterfering repair pathways.

### Meiotic DSBs are elevated in the pericentromeres of non-CG/H3K9me2 mutants

During *A. thaliana* meiosis, SPO11-1 acts with SPO11-2 and MTOPVIB to generate DSBs, which can undergo inter-homolog repair to form crossovers (Grelon et al. 2001; Hartung et al. 2007; Vrielynck et al. 2016). SPO11 enzymes are related to topoisomerase-VI transesterases and become covalently bound to ∼20- to 50-base target site oligonucleotides during DSB formation (Keeney and Kleckner 1995; Pan et al. 2011; Lange et al. 2016). We have purified and sequenced *A. thaliana* SPO11-1-oligos in order to map patterns of meiotic DSBs genome-wide, using a complementing *SPO11-1-Myc spo11-1* line (Choi et al. 2018). In order to profile meiotic DSBs in H3K9me2/non-CG DNA methylation mutants, we generated a *SPO11-1-Myc spo11-1 kyp suvh5 suvh6* line and used this to purify and sequence SPO11-1-oligos (Supplemental Table S16). The coverage of combined unique and multiple mapped SPO11-1-oligo reads were normalized by library size. Replicate libraries showed significant correlation at multiple scales (Supplemental Table S17). For example, Pearson's *r* values between replicate libraries at the 10-kb scale were between 0.91 and 0.99 (Supplemental Table S17). Further normalization was performed using a single-ended Col genomic DNA library with reads trimmed to 50 base pairs, which were aligned as for SPO11-1-oligos, and then used to calculate log_2_(SPO11-1-oligo/gDNA) values. Finally, *z*-score standardization was applied, such that scores represent the signed number of standard deviations from the mean, and these values were used for downstream analysis.

**Figure 5.**
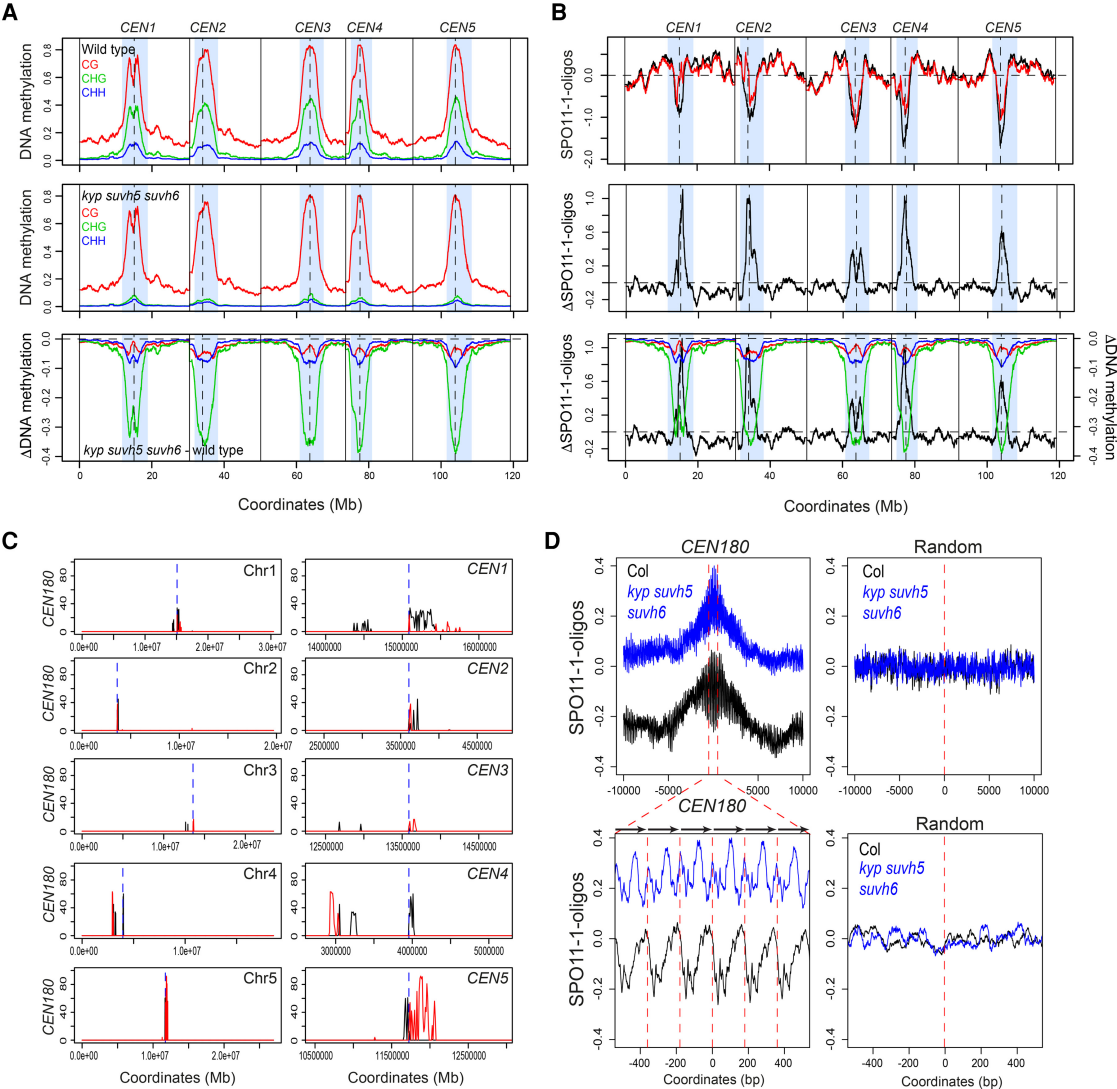
Elevated SPO11-1-oligonucleotides levels in centromeres of *kyp suvh5 suvh6* H3K9me2 mutants. (*A*) Plots of the *A. thaliana* chromosomes on a continuous *x*-axis are shown. Analysis of DNA methylation frequency in CG (red), CHG (green), and CHH (blue) from published data in wild type (Col) (*top*) or *kyp suvh5 suvh6* (*middle*) (Stroud et al. 2013). A *kyp suvh5 suvh6* minus wild type differential (ΔDNA methylation) plot is also shown (*bottom*). Vertical dotted lines indicate the position of the centromere assembly gaps and vertical solid lines indicate telomeres. The pericentromeres, defined by higher than average DNA methylation, are indicated by light blue shading. (*B*) Plots showing log_2_(SPO11-1-oligos/gDNA) (SPO11-1-oligos) in wild type (Col, black) and *kyp suvh5 suvh6* (red) (*top*), in addition to the *kyp suvh5 suvh6-*wild type differential (ΔSPO11-1-oligos) (*middle*), which is also overlaid with the ΔDNA methylation from *A* (*bottom*). Plots are annotated as in *A*. (*C*) Plots of the *A. thaliana* chromosomes showing the density of *CEN180* repeats on forward (black) and reverse (red) strands. The TAIR10 centromeric assembly gaps are shown by the dotted blue line. The plots on the *left* show whole chromosomes, whereas the plots on the *right* show a close-up of the regions surrounding the centromere assembly gaps. (*D*) The density of log_2_(SPO11-1-oligos/gDNA) (SPO11-1-oligos) was analyzed in ±10-kb windows surrounding matches to the *CEN180* consensus in wild type (black) or *kyp suvh5 suvh6* (blue). The *lower* plot corresponds to a blow-up of a 1-kb window around the center of the *upper* plot. The lower plot highlights the approximate positions of *CEN180* repeats using arrows and red dotted lines. Also shown is an identical analysis performed for the same number of randomly chosen sites.

To analyze chromosome-scale patterns, SPO11-1-oligo levels were calculated in 10-kb adjacent windows and plotted along the chromosomes using a rolling average. We also calculated a SPO11-1-oligo differential (Δ) by subtracting wild-type from *kyp suvh5 suvh6* values. We observed that the centromeric and pericentromeric regions showed a striking increase in SPO11-1-oligos in *kyp suvh5 suvh6* (Fig. 5A,B). We similarly calculated mean CG, CHG, and CHH methylation levels in 10-kb windows and calculated *kyp suvh5 suvh6* differential values in the same way, using published data (Stroud et al. 2013). The *kyp suvh5 suvh6* mutant shows strong reduction of CHG and CHH methylation in the pericentromeric regions, which was significantly correlated with the SPO11-1-oligo Δ differential (Pearson's CHG *r* = −0.751, *P* <2.2 × 10^−16^, CHG *r* = −0.737, *P* = <2.2 × 10^−16^) (Fig. 5A,B). The increase in SPO11-1-oligos in *kyp suvh5 suvh6* occurred more strongly within the genetically defined centromeres, compared with the crossover changes in *cmt3*, which occurred most strongly within the adjacent pericentromeres (Figs. 2D,F, 5A,B). Hence, while both meiotic DSBs and crossovers increase in H3K9me2/non-CG mutant heterochromatin, they were elevated in adjacent centromeric and pericentromeric regions, respectively.

We next analyzed DSB frequency around copies of the *CEN180* satellite repeat, which are found in proximity to the centromeres (Fig. 5C). Each *A. thaliana* chromosome sequence contains a centromere gap, which contains megabase arrays of *CEN180* repeats (Copenhaver et al. 1999; Kumekawa et al. 2000; Ito et al. 2007; Maheshwari et al. 2017). Further matches to the *CEN180* consensus flank these gaps, and we identified 3397 repeats in the Col reference genome that generally occur in tandemly repeated arrays (Fig. 5C). SPO11-1-oligo density was analyzed in 20-kb windows around these repeat positions and compared to the same number of randomly chosen windows, in wild type and *kyp suvh5 suvh6* (Fig. 5D). Consistent with the SPO11-1-oligo increase in centromeric regions at the chromosome scale (Fig. 5A), we observed a pronounced increase in SPO11-1-oligo density within the *CEN180* repeats at the fine-scale in *kyp suvh5 suvh6* (Fig. 5D). The pattern of SPO11-1-oligos within the *CEN180* repeat units is also altered in *kyp suvh5 suvh6* (Fig. 5D). Together, this shows that meiotic DSBs increase in the *CEN180* repeats in H3K9me2 mutant backgrounds. A relatively small number (141) of transposons are transcriptionally up-regulated in *kyp suvh5 suvh6* mutants and associated with decreased non-CG methylation (Stroud et al. 2012, 2013). Some of these elements were associated with elevated SPO11-1-oligonucleotides in *kyp suvh5 suvh6* mutants, but others were not (Supplemental Fig. S3), indicating that transcriptional activation is not strictly coupled to up-regulation of meiotic SPO11-1-oligo formation at *A. thaliana* transposons.

### Transposon insertions into *CMT3* appear to induce meiotic recombination

In a large scale screen for de novo insertions of the nonautonomous maize transposable element *Dissociation* (*Ds*) introduced into *A. thaliana*, a transgene strongly expressing the *Activator* (*Ac*) transposase was crossed to plants containing *Ds* “launch-pads,” triggering *Ds* transposition in clonal cell lineages within F_1_ plants (Sundaresan et al. 1995). As *Ds* elements are known to preferentially transpose to linked sites (Jones et al. 1990; Bancroft and Dean 1993), a positive–negative selection scheme was implemented in F_2_ progeny to select against the *Ds* launch-pad and for transposed *Ds* (Sundaresan et al. 1995). In this way, recovery of F_2_ transpositions was dependent on recombination between the transposed *Ds* and the launch-pad and should be mostly unlinked.

**Figure 6.**
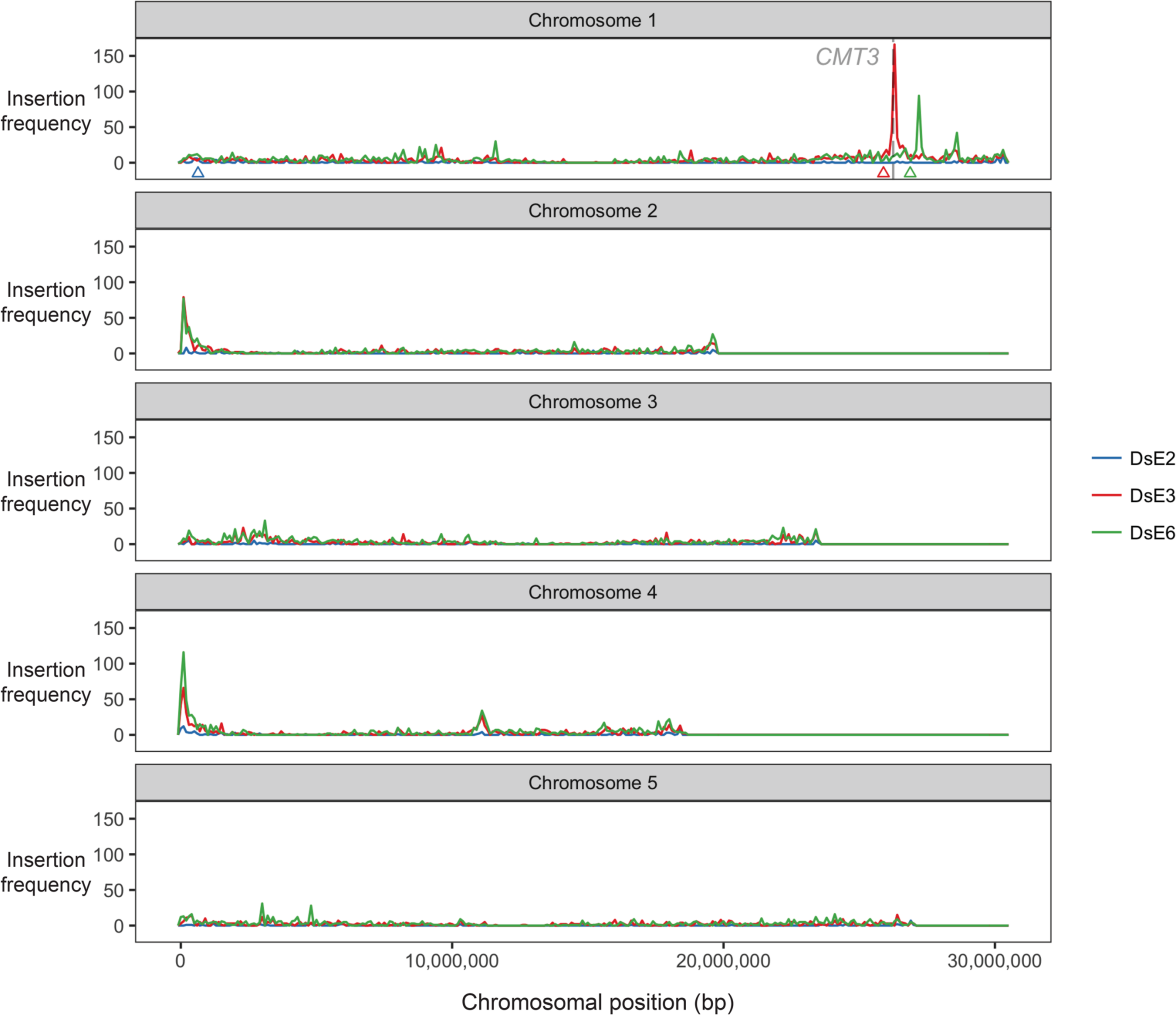
Transposon insertions in *CMT3* appear to induce recombination. Enhancer trap DNA transposons (*DsE*) based on the maize transposable element *Dissociation* (*Ds*) were introduced into the *A. thaliana* genome on a T-DNA with a negative selectable marker (Sundaresan et al. 1995). Three independent *DsE* launch-pads were mapped to Chromosome 1: *DsE2* (633,819 bp, blue triangle), *DsE3* (25,893,665 bp, red triangle), and *DsE6* (26,877,449 bp, green triangle). After introducing *Activator* transposase, 9622 transpositions of *DsE* were isolated in F_2_ progeny by selecting for the transposon, but against the launch-pad, to select against linked transpositions which would otherwise be highly favored. New *DsE* insertions were mapped by sequencing flanking DNA. Transpositions from each Chromosome 1 launch-pad are displayed in 100-kb bins, and most were unlinked, including two hotspots corresponding to nucleolar organizer regions at the ends of Chromosomes 2 and 4. However, two much sharper insertions hotspots, on Chromosome 1, were specific for closely linked launch-pads, *DsE3* (red triangle) and *DsE6* (green triangle), respectively. The hotspot immediately distal to *DsE3* (∼4% of transpositions launched from *DsE3*) was in and immediately around the *CMT3* gene (26,248,318–26,253,585 bp).

Three launch-pads on Chromosome 1, *DsE2*, *DsE3*, and *DsE6* (Fig. 6), were used to generate 9622 independent transpositions, which were mapped using TAIL-PCR (Springer et al. 1995). Unexpectedly, we recovered a dramatic enrichment of homozygous and heterozygous insertions into and nearby the *CMT3* locus (214 out of 4044 insertions in total), but only when *Ds* elements were launched from a closely linked (∼360 kb) centromere-proximal locus on Chromosome 1 (*DsE3*) and not from a centromere-distal locus (*DeE6*) located a comparable distance from *CMT3* (∼624 kb) (Fig. 6). One possible explanation was that flowers carrying homozygous *cmt3* insertions arose by transposition into *CMT3*, followed by imprecise excision and mitotic recombination. Subsequent meiotic recombination between heterozygous transposed *Ds* elements and the launch-pad might be enhanced in *cmt3* anthers, leading to increased recovery of F_2_ “transposant” progeny carrying insertions near *CMT3*. *Ac* elements in maize and *Ds* elements in *A. thaliana* can stimulate mitotic recombination between flanking repeats (Athma and Peterson 1991; Xiao et al. 2000), but the excision/recombination scenario envisioned here has not been previously observed. Further work will be required to investigate the role of mitotic and meiotic recombination in the enhanced recovery of *Ds* insertions near *CMT3*, and the extent that this might be caused by disruption of the non-CG DNA methylation/H3K9me2 pathway is currently unknown.

## Discussion

Meiotic crossover in proximity to centromeres has been associated with chromosome missegregation and aneuploidy in fungi and animals (Koehler et al. 1996; Lamb et al. 1996; Rockmill et al. 2006). Hence, suppression of crossovers within centromeres is thought to play an important role in maintaining fidelity of genome transmission during meiosis. However, abundant evidence for recombination-associated polymorphism in centromeric repeats exists, consistent with the effects of replication slippage, unequal crossover, and gene conversion (Ma and Bennetzen 2006; Nambiar and Smith 2016; Wolfgruber et al. 2016). For example, maize centromeric *CRM* retrotransposons have been observed to undergo meiotic gene conversion but not crossover (Shi et al. 2010), which indicates initiation of meiotic DSBs but downstream inhibition of recombination leading to crossover. We also observe evidence for SPO11-1-dependent DSBs within the *A. thaliana CEN180* repeats, meaning that meiotic recombination may contribute to polymorphism within centromeric satellite repeat arrays in this species (Ito et al. 2007; Maheshwari et al. 2017).

As centromeric DSBs increase in *kyp suvh5 suvh6* mutants, this demonstrates that epigenetic information, including H3K9me2 and non-CG DNA methylation, plays important roles in suppressing initiation of meiotic recombination in these regions. However, our data also reveal complexity in how chromatin shapes meiotic recombination around plant centromeres. First, while the genetically defined centromeric regions show increased DSBs in *kyp suvh5 suvh6*, in *cmt3* we observed that crossovers were most elevated in adjacent pericentromeric regions. An important distinction between these experiments is that SPO11-1-oligos were mapped in a Col/Col homozygous background, whereas mapping crossover necessitates use of polymorphic Col/Ler hybrids. In a Col/Ler hybrid context, centromeric crossovers are likely to be additionally suppressed by structural polymorphisms. For example, the inhibitory effect of structural polymorphism is evident within the ∼1.17-Mb heterochromatic knob inversion on Chromosome 4, which is suppressed for crossovers in both wild-type and *cmt3* populations (Fig. 2D; Fransz et al. 2000, 2016). Following DSB formation, resection occurs to generate 3′ single-stranded DNA that can perform strand invasion of homologous chromosomes (Keeney and Neale 2006). Inter-homolog recombination downstream from strand invasion is sensitive to heterology between the recombining chromosomes, which can have a local inhibitory effect on crossover formation and instead promote noncrossover repair and gene conversion (Dooner 1986; Borts and Haber 1987). *A. thaliana* centromeric regions exhibit extensive structural variation, including within Gypsy retroelements (Kumekawa et al. 2000; Ito et al. 2007; Quadrana et al. 2016; Stuart et al. 2016), which may therefore suppress centromeric crossover formation downstream from inter-homolog strand invasion, despite activation of meiotic DSBs in non-CG/H3K9me2 mutants. It is also possible that additional chromatin or epigenetic features enriched within the centromeres suppress crossover repair. For example, the kinetochore, CENH3 nucleosomes, or further heterochromatic marks such as H2A.W may be differentially enriched within the centromeric versus pericentromeric regions and cause inhibition of crossover maturation (Yelagandula et al. 2014; Vincenten et al. 2015).

An important question raised by our study is why pericentromeric crossover frequency increases in the H3K9me2/non-CG pathway mutants reported here, but not in *met1* and *ddm1* where reduced pericentromeric crossover frequency is observed (Colomé-Tatché et al. 2012; Melamed-Bessudo and Levy 2012; Mirouze et al. 2012; Yelina et al. 2012, 2015), despite both *kyp suvh5 suvh6* and *met1* showing increased SPO11-1-oligos in proximity to centromeres (Choi et al. 2018). MET1 and DDM1 play major roles in the maintenance of DNA methylation in the CG context. However, their molecular roles in other respects are distinct: (1) Non-CG DNA methylation is reduced in *ddm1* to a greater extent than *met1* (Stroud et al. 2013); (2) gene body methylation is eliminated in *met1* but not *ddm1* (Stroud et al. 2013); and (3) H3K9me2 is reduced more strongly in *ddm1* compared with *met1* (Gendrel et al. 2002; Deleris et al. 2012). Therefore, we postulate that their common feature, loss of CG methylation within heterochromatin, alters progression and maturation of the meiotic recombination pathway, such that crossovers are favored in the chromosome arms, at the expense of the pericentromeres (Colomé-Tatché et al. 2012; Melamed-Bessudo and Levy 2012; Mirouze et al. 2012; Yelina et al. 2012, 2015). In contrast, H3K9me2/non-CG pathway mutants activate recombination in the heterochromatic regions such that maturation of crossovers in the pericentromeres is increased. Indeed, distinctions in recombination phenotype are consistent with the different effects on transcription, transposition, and chromosomal conformation associated with loss of CG versus non-CG DNA methylation maintenance pathways (Miura et al. 2001; Singer et al. 2001; Kato et al. 2003; Lippman et al. 2004; Zhang et al. 2006; Henderson and Jacobsen 2008; Lister et al. 2008; Colomé-Tatché et al. 2012; Melamed-Bessudo and Levy 2012; Mirouze et al. 2012; Stroud et al. 2012, 2013, 2014; Feng et al. 2014; Yelina et al. 2015).

We propose that, while both CG and non-CG DNA methylation inhibit centromeric meiotic DSBs (Choi et al. 2018), only non-CG methylation and/or H3K9me2 inhibit crossovers. In agreement with this idea, euchromatic crossover hotspots in *A. thaliana* can be silenced by RNA-directed DNA methylation associated with gain of H3K9me2 and both CG and non-CG methylation (Yelina et al. 2015). Furthermore, in fission yeast, arrested recombination intermediates accumulate strongly in wild-type heterochromatin, but not in *clr4* and *rik1* (*Recombination In K*) heterochromatin, which lose H3K9me2 and undergo mitotic and meiotic recombination (Ellermeier et al. 2010; Zaratiegui et al. 2011). Mouse *dnmt3l* mutants also have altered DNA methylation and chromatin signatures and increased DSB initiation within retrotransposons, which is associated with meiotic catastrophe and infertility (Zamudio et al. 2015). In contrast, *A. thaliana* H3K9me2 and DNA methylation mutants are fully fertile, despite increased recombination initiation in the centromeric regions, suggesting that increased meiotic DSBs in transposons do not, per se, cause infertility. Suppression of heterochromatic recombination is a major barrier to introducing genetic diversity in crop plants like maize and wheat, where the majority of the chromosome is composed of pericentromeric heterochromatin and yet contains important genetic variation in functional genes and traits (Gore et al. 2009; Choulet et al. 2014). Therefore, an exciting prospect will be to modulate H3K9me2 and non-CG DNA methylation to unlock pericentromeric crossovers in crop breeding programs.

## Methods

### Plant material

*A. thaliana* plants were grown under long-day conditions (16 h light/8 h dark, at 150 µmol light intensity) at 20°C. We used the following mutant alleles: *kyp-6* (SALK_041474) (Chan et al. 2006), *cmt3-11* (SALK_148381) (Chan et al. 2006), *cmt3-7* (Lindroth et al. 2001), *kyp suvh5 suvh6* (SALK_041474, GK-263C05, SAIL_1244_F04) (Johnson et al. 2008), *drm1-2 drm2-2* (SALK_031705, SALK_150863) (Chan et al. 2006), *drm1-2 drm2-2 cmt3-11* (SALK_031705, SALK_150863, SALK_148381) (Chan et al. 2006), *cmt2-3* (SALK_012874) (Stroud et al. 2014), *ddm1* (SALK_000590), *zip4-2* (SALK_068052) (Chelysheva et al. 2007), and *fancm-1* (EMS point mutant) (Crismani et al. 2012). The centromeric FTLs *CTL5.11* and *LTL5.4* were obtained from the Traffic line population (Wu et al. 2015).

### Mapping of *Ds* insertion sites

Generation of the *Ds* transposant lines was previously reported. *Ds* insertion sites were amplified by TAIL-PCR (Springer et al. 1995; Sundaresan et al. 1995).

### Measuring crossovers using fluorescent pollen and seed

Crossover scoring using the pollen FTL *CEN3* was performed by flow cytometry, as previously reported (Yelina et al. 2013). Crossover scoring using seed FTLs (*420*, *CTL5.11*, *LTL5.4*) was performed by fluorescent imaging, as previously reported (Ziolkowski et al. 2015). Statistical analysis of fluorescent count data was performed as described (Yelina et al. 2015; Ziolkowski et al. 2015).

### Genotyping-by-sequencing

Illumina sequencing libraries were constructed in 96-well format, as previously reported (Rowan et al. 2015; Yelina et al. 2015), with the following minor modifications. DNA was extracted from three rosette leaves of 5-wk-old plants and 150 ng of DNA used as input for each library. DNA shearing was carried out for 20 min at 37°C with 0.4 units of DNA Shearase (Zymo Research). Each set of 96 libraries was sequenced on an Illumina NextSeq 500 (300-cycle Mid Output run). Sequencing data was analyzed to identify crossovers as previously reported, using the TIGER pipeline (Rowan et al. 2015; Yelina et al. 2015). Three hundred eighty-four *cmt3-11* × *cmt3-7* F_2_ individuals were sequenced. Wild-type crossovers (CO) were mapped by sequencing 245 Col × Ler F_2_ individuals, which were combined with data from 192 F_2_ individuals (Choi et al. 2016).

The coordinates of crossover intervals called by TIGER were used for subsequent analysis. Centromeres were genetically defined as contiguous regions flanking the TAIR10 centromeric assembly gap that show an absence of crossovers in wild type (Copenhaver et al. 1999; Giraut et al. 2011; Salomé et al. 2012). We define the pericentromeric regions as regions flanking the centromeres with higher than chromosome average levels of DNA methylation. The euchromatic arms constitute the remainder of the chromosomes, from the telomeres to the pericentromeres (Supplemental Table S9). Crossovers (midpoints) were counted in these regions and compared to those expected at random according to physical distance and χ^2^ tests performed (Supplemental Table S10).

For chromosomal plots of crossovers and DNA methylation, crossovers were tallied in 10-kb windows along the chromosomes, and crossover/10-kb values were then divided by the number of F_2_ individuals analyzed for each genotype. For DNA methylation, mean values were calculated in 10-kb windows. Finally, for both crossovers and DNA methylation data, a rolling mean calculation was applied to smooth the data prior to plotting using the R function filter (R Core Team 2012). The crossover and DNA methylation differentials were calculated by subtracting wild-type from *cmt3* values.

For telomere-centromere analysis, chromosome arms were first oriented such that each began at the telomere and ended at the centromere. The arms were then divided into windows representing 1% of their proportional lengths and crossovers assigned to these windows. Crossover values were divided by the number of F_2_ individuals analyzed for each genotype. These values were averaged across all chromosome arms and then plotted along the telomere-centromere axis with smoothing applied using the R function smooth.spline. For DNA methylation analysis along telomere-centromere axes, values were first calculated in 10-kb windows along the chromosomes. Chromosome arms were then oriented such that each began at the telomere and ended at the centromere and divided into windows representing 0.5% of their proportional lengths. Methylation values were then averaged across all chromosome arms and plotted along the telomere-centromere axis.

### SPO11-1-oligonucleotide sequencing

A detailed protocol and analysis methodology are provided in an accompanying manuscript (Choi et al. 2018). A complementing *SPO11-1-Myc spo11-1* line was crossed with *kyp suvh5 suvh6* triple mutants and *SPO11-1-Myc spo11-1 kyp suvh5 suvh6* plants identified for analysis. The *CEN180* consensus sequence (5′-AACCTTCTTCTTGCTTCTCAAAGCTTTCATGGTGTAGCCAAAGTCCATATGAGTCTTTGGCTTTGTGTCTTCTAACAAGGAAACACTACTTAGGCTTTTAAGATCCGGTTGCGGTTTAAGTTCTTATACTCAATCATACACATGACATCAAGTCATATTCGACTCCAAAACACTAACC-3′) was matched to the TAIR10 reference sequence using the R function matchPattern with max.mismatch set to 90. The coverage value of normalized SPO11-1-oligonucleotides was analyzed in 20-kb windows around *CEN180* matches and compared with analysis of the same number of randomly chosen positions.

### Meiotic immunocytology

Chromosome spreads of *A. thaliana* pollen mother cells and immunostaining of ASY1 and H3K9me2 were performed using fresh buds, as described (Armstrong et al. 2002). Immunostaining of MLH1 was performed on acetic acid chromosome spreads on fixed buds, as described (Chelysheva et al. 2010). The following antibodies were used: α-ASY1 (rabbit, 1/500 dilution, gift from Chris Franklin [University of Birmingham]) (Armstrong et al. 2002), α-H3K9me2 (mouse, 1/200 dilution, Abcam, ab1220), and α-MLH1 (rabbit, 1/200 dilution, gift from Mathilde Grelon [INRA, Versailles]) (Chelysheva et al. 2010). Microscopy was conducted using a DeltaVision Personal DV microscope (Applied Precision/GE Healthcare) equipped with a CDD CoolSNAP HQ2 camera (Photometrics). Image capture was performed using softWoRx software version 5.5 (Applied Precision/GE Healthcare). All slides within an experiment (e.g., Col, *cmt3*, and *kyp suvh5 suvh6*) were prepared alongside one another and images captured using the same exposure time. The staining pattern of ASY1 and DNA was used to identify cells at leptotene, zygotene, or pachytene stage (Sanchez-Moran et al. 2007). Wild-type (Col) cells were first analyzed and a threshold pixel intensity value identified that removed background signal. This threshold was then applied to all images prior to further processing. Individual cell images were acquired as Z-stacks of 16 optical sections of 0.25 µm each, and the maximum intensity projection of the cell was reconstructed using ImageJ, as described (Lambing et al. 2015). The boundaries of each cell were manually defined and the total signal intensity within the cell measured. An adjacent region outside of the cell was used to measure mean background intensity and this value used to subtract from the within-cell intensity. The same methods were used for analysis of meiotic and somatic cells.

## Data access

All data from this study have been submitted to ArrayExpress (https://www.ebi.ac.uk/arrayexpress/): SPO11-1-oligonucleotide sequencing data in wild type and *kyp suvh5 suvh6* under accession number E-MTAB-5041; control libraries for SPO11-1-oligonucleotide sequencing under accession number E-MTAB-6257; and GBS crossover data from wild type and *cmt3* populations under accession numbers E-MTAB-4657, E-MTAB-5476, and E-MTAB-6577 (GBS).

## Supplementary Material

Supplemental Material
