# Supplementary material for: Epigenetic activation of meiotic recombination near Arabidopsis thaliana centromeres via loss of H3K9me2 and non-CG DNA methylation
Source: Genome Res. 2018 Apr;28(4):519–31. doi: 10.1101/gr.227116.117 (PMC5880242; doi:10.1101/gr.227116.117)
Supplement: Supplemental Material [file supp_28_4_519__index.html]

Epigenetic activation of meiotic recombination near Arabidopsis thaliana centromeres via loss of H3K9me2 and non-CG DNA methylation — Supplemental Material 

# Epigenetic activation of meiotic recombination near *Arabidopsis thaliana* centromeres via loss of H3K9me2 and non-CG DNA methylation

## Supplemental Material

- Supplemental\_Fig\_S1.pdf
- Supplemental\_Fig\_S2.pdf
- Supplemental\_Fig\_S3.pdf
- Supplemental\_Material.docx
