# Supplementary figures and images for: Epigenetic activation of meiotic recombination near Arabidopsis thaliana centromeres via loss of H3K9me2 and non-CG DNA methylation
Source: Genome Res. 2018 Apr;28(4):519–31. doi: 10.1101/gr.227116.117 (PMC5880242; doi:10.1101/gr.227116.117)

A

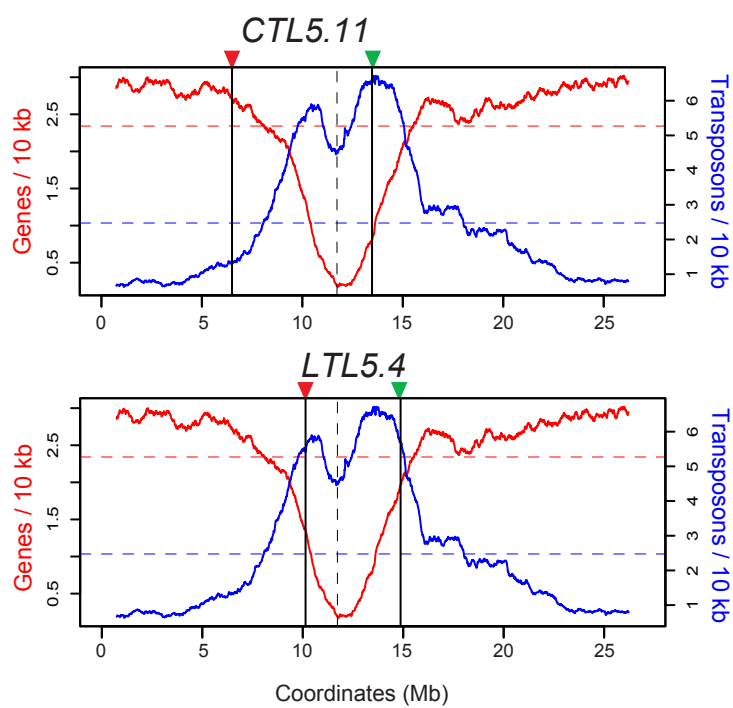

B

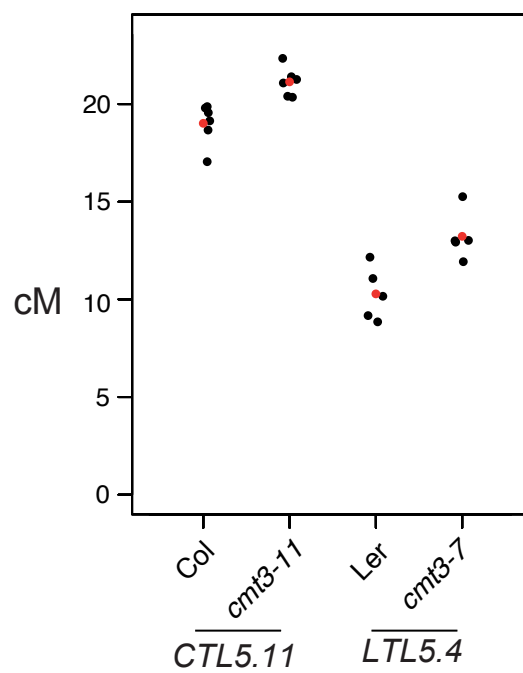

Supplement: Supplemental Material [file supp_gr.227116.117_Supplemental_Fig_S1.pdf]

A

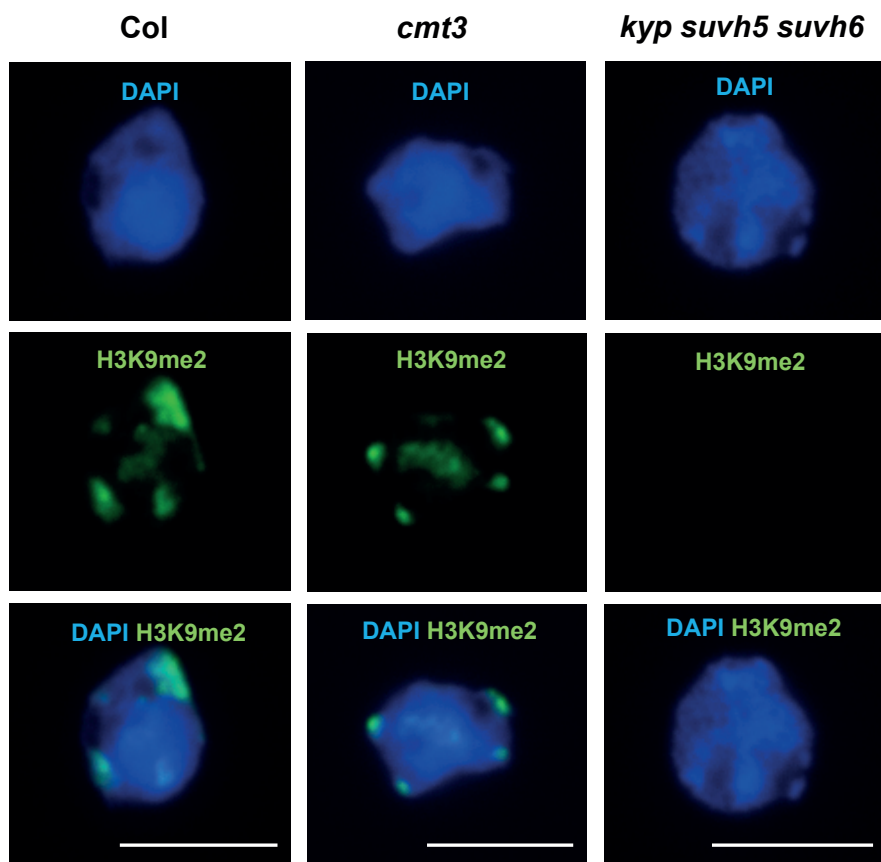

B

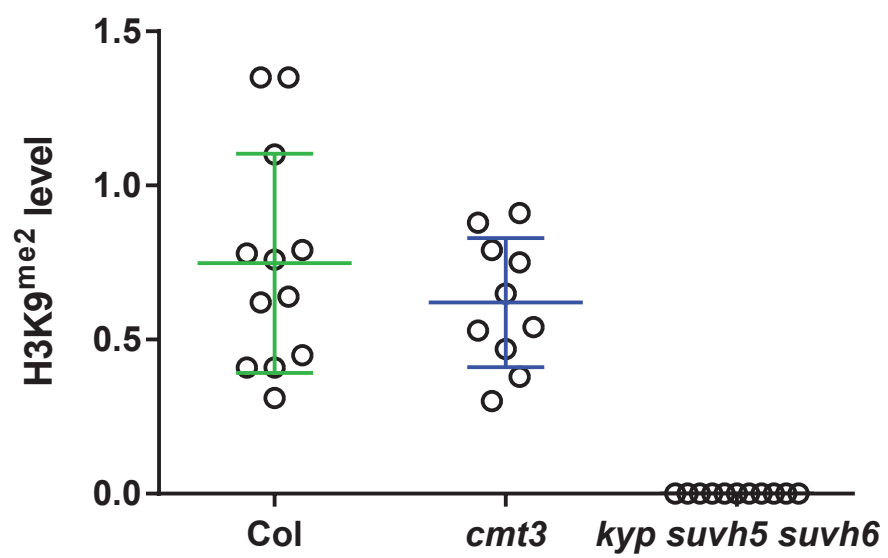

Supplement: Supplemental Material [file supp_gr.227116.117_Supplemental_Fig_S2.pdf]

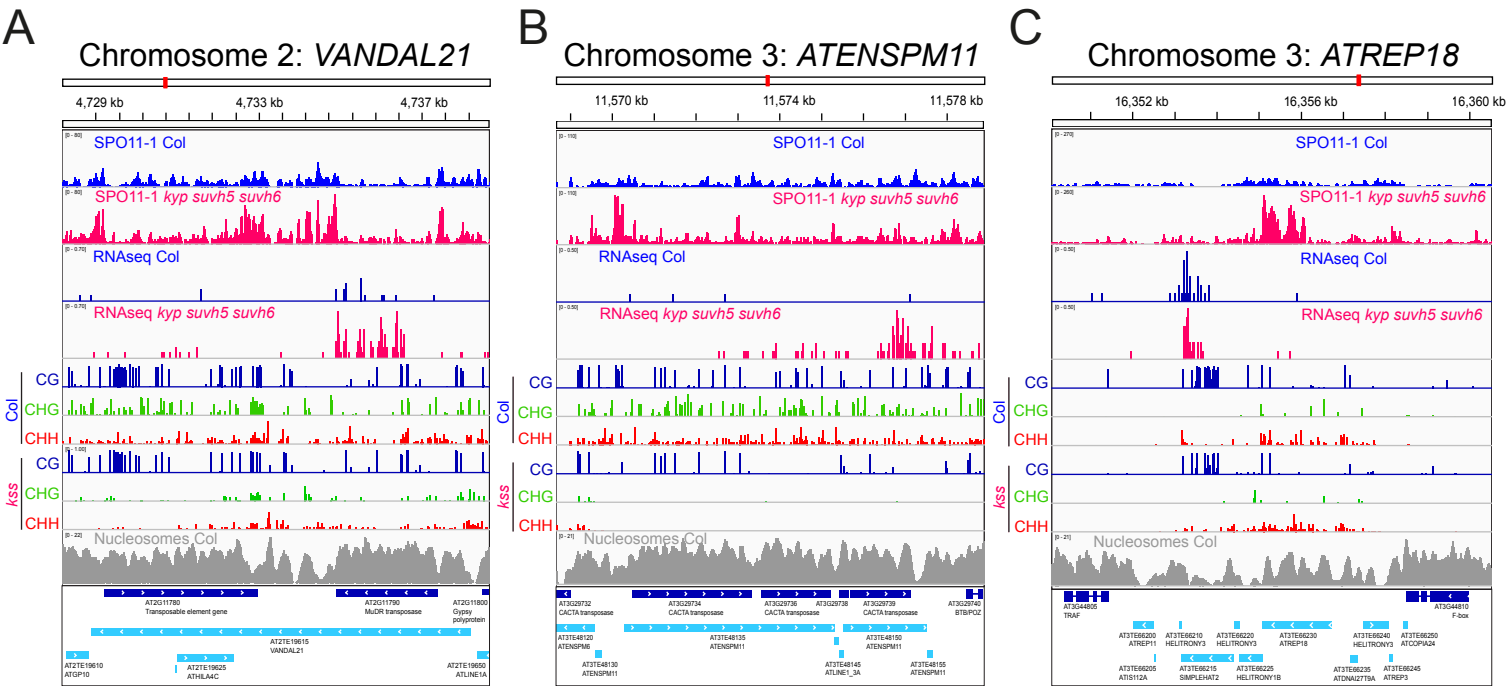

Supplement: Supplemental Material [file supp_gr.227116.117_Supplemental_Fig_S3.pdf]
