# Supplementary material for: Epigenetic activation of meiotic recombination near Arabidopsis thaliana centromeres via loss of H3K9me2 and non-CG DNA methylation
Source: Genome Res. 2018 Apr;28(4):519–31. doi: 10.1101/gr.227116.117 (PMC5880242; doi:10.1101/gr.227116.117)
Supplement: Supplemental Material [file supp_gr.227116.117_Supplemental_Material.docx]

**Epigenetic activation of meiotic recombination in *Arabidopsis* centromeres via loss of H3K9me2 and non-CG DNA methylation**

**SUPPLEMENTAL INFORMATION**

**CONTENTS**

Supplemental Figures S1-S3

Supplemental Tables S1-S19

**Supplemental Figure S1. Increase of Chromosome 5 pericentromeric recombination by *cmt3* mutations in Col and Ler accessions. (A)** Gene (red) and transposon (blue) density along Chromosome 5. Mean values are indicated by the horizontal dotted lines, and the centromere by the vertical dotted line. The location of the *CTL5.11* and *LTL5.4* FTL T-DNAs are indicated by black vertical lines. (**B)** Crossover frequency (cM) in the *CTL5.11* and *LTL5.4* intervals in wild type and *cmt3-11* or *cmt3-7* backgrounds.

**Supplemental Figure S2. Immunostaining for H3K9me2 in somatic nuclei in wild type, *cmt3* and *kyp suvh5 suvh6*. (A)** Wild type (Col) and *kyp suvh5 suvh6* somatic cells were immunostained for ASY1 (red) and H3K9me2 (green) and stained for DNA (DAPI, blue). The scale bar shows 10μM. (**B)** Quantification of H3K9me2 immunostaining signal in wild type (Col) and *kyp suvh5 suvh6* somatic cells.

**Supplemental Figure S3. Fine-scale changes to transcription, SPO11-1-oligonucleotides and DNA methylation at transposons in *kyp suv5 suvh6.* (A)** Plot of SPO11-1 and RNAseq (Stroud et al. 2012) from wild type (Col, blue) and *kyp suvh5 suvh6* (red) around the *VANDAL21* DNA transposon. Also plotted is DNA methylation in CG (blue), CHG (green) and CHH (red) sequence contexts from wild type (Col) and *kyp suvh5 suvh6* (Stroud et al. 2013), nucleosome occupancy from wild type (Choi et al. 2016) and gene and transposon annotation (Buisine et al. 2008). **(B)** As for (A), but showing the *ATENSPM11* DNA transposon. **(C)** As for (A), but showing a region around *ATREP18*.

**Supplemental Tables**

**Supplemental Table S1. Fluorescent pollen count data for the *CEN3* FTL interval in wild type, H3K9me2 and DNA methylation mutants.** *CEN3* genetic distance is calculated as cM = 100 × 2 × R_6_ / (R_2_ – (R_5_ – R_4_))*.* Where R_2_ is the total number of pollen analysed, R_4_ is the number of eYFP and RFP positive pollen, R_5_ is the number of eYFP and RFP negative pollen and R_6_ is the number of eYFP positive pollen (Ziolkowski et al. 2015a; Yelina et al. 2015). *CEN3* data for *met1/+* and *met1* are reproduced from (Yelina et al. 2015). The number of recombinant (R_6_) and non-recombinant (R_4_) counts for wild type and mutant genotypes were used to construct 2×2 contingency tables and *X^2^* tests performed to test for significant differences.

| Genotype | Total (R_2_) | eYFP/RFP (R_4_) | No colour (R_5_) | eYFP (R_6_) | cM | *X^2^* *P* |
| --- | --- | --- | --- | --- | --- | --- |
| Wild type (Col) | 10570 | 3438 | 5865 | 503 | 12.35 |  |
| Wild type (Col) | 13631 | 5130 | 6918 | 698 | 11.79 |  |
| Wild type (Col) | 14478 | 4488 | 8415 | 548 | 10.39 |  |
| Wild type (Col) | 7774 | 3031 | 3845 | 378 | 10.86 |  |
| Wild type (Col) | 7420 | 2777 | 3795 | 363 | 11.34 |  |
| Wild type (Col) | 6858 | 2726 | 3333 | 353 | 11.29 |  |
| Wild type (Col) | 6665 | 2586 | 3312 | 325 | 10.94 |  |
| Wild type (Col) | 7864 | 3173 | 3819 | 399 | 11.06 |  |
| Wild type (Col) | 4070 | 1422 | 2189 | 187 | 11.32 |  |
| Wild type (Col) | 7460 | 2665 | 3943 | 329 | 10.64 |  |
| Wild type (Col) | 12526 | 4459 | 6527 | 585 | 11.19 |  |
| Wild type (Col) | 15471 | 5948 | 7557 | 820 | 11.83 |  |
| Wild type (Col) | 1920 | 593 | 1116 | 67 | 9.59 |  |
| Wild type (Col) | 11407 | 4029 | 5956 | 541 | 11.41 |  |
| Wild type (Col) | 15900 | 5615 | 8178 | 785 | 11.77 |  |
| Wild type (Col) | 7302 | 2610 | 3719 | 358 | 11.56 |  |
| Wild type (Col) | 1755 | 573 | 954 | 76 | 11.06 |  |
| Wild type (Col) | 9565 | 3198 | 5107 | 464 | 12.12 |  |
| Wild type (Col) | 9384 | 2670 | 5436 | 385 | 11.63 |  |
| Wild type (Col) | 15463 | 5265 | 7924 | 705 | 11.01 |  |
| Wild type (Col) | 10617 | 3907 | 5505 | 482 | 10.69 |  |
| Wild type (Col) | 14089 | 5321 | 7048 | 681 | 11.02 |  |
| Wild type (Col) | 12193 | 3402 | 7129 | 461 | 10.89 |  |
| Wild type (Col) | 14733 | 5232 | 7408 | 727 | 11.58 |  |
| Wild type (Col) | 13010 | 3808 | 7510 | 529 | 11.37 |  |
| Wild type (Col) | 15787 | 5006 | 8805 | 635 | 10.59 |  |
| Wild type (Col) | 16641 | 5071 | 9562 | 659 | 10.85 |  |
| Wild type (Col) | 11784 | 3653 | 6710 | 430 | 9.85 |  |
| Wild type (Col) | 12501 | 4635 | 6410 | 571 | 10.65 |  |
| Wild type (Col) | 12115 | 4378 | 6377 | 535 | 10.58 |  |
| Wild type (Col) | 13542 | 4738 | 7267 | 616 | 11.19 |  |
| Wild type (Col) | 9129 | 3265 | 4865 | 397 | 10.55 |  |
| Wild type (Col) | 10797 | 4078 | 5476 | 513 | 10.92 |  |
| Wild type (Col) | 10926 | 3820 | 5861 | 504 | 11.34 |  |
| Wild type (Col) | 12737 | 5172 | 6030 | 654 | 11.01 |  |
| Wild type (Col) | 6671 | 2387 | 3538 | 325 | 11.78 |  |
| Wild type (Col) | 10928 | 4380 | 5282 | 573 | 11.43 |  |
| Wild type (Col) | 8461 | 3194 | 4257 | 419 | 11.33 |  |
| Wild type (Col) | 15286 | 6157 | 7345 | 764 | 10.84 |  |
| Wild type (Col) | 16257 | 5540 | 9001 | 774 | 12.10 |  |
| Wild type (Col) | 14385 | 5166 | 7660 | 723 | 12.16 | n.d. |
| *KYP/kyp suvh5 suvh6* | 7936 | 2973 | 4061 | 398 | 11.62 |  |
| *KYP/kyp suvh5 suvh6* | 7119 | 2526 | 3836 | 318 | 10.95 |  |
| *KYP/kyp suvh5 suvh6* | 9034 | 3559 | 4391 | 432 | 10.53 |  |
| *KYP/kyp suvh5 suvh6* | 3367 | 1113 | 1867 | 134 | 10.26 | 0.18 |
| *suvh5 suvh6* | 7828 | 2978 | 3984 | 376 | 11.02 |  |
| *suvh5 suvh6* | 4411 | 1276 | 2771 | 177 | 12.14 | 0.84 |
| *cmt2* | 16173 | 5157 | 8795 | 628 | 10.02 |  |
| *cmt2* | 4710 | 1290 | 2720 | 188 | 11.46 |  |
| *cmt2* | 17153 | 5544 | 9403 | 798 | 12.01 |  |
| *cmt2* | 26549 | 8426 | 14845 | 1123 | 11.16 | 0.35 |
| *drm1 drm2* | 24691 | 7484 | 14277 | 1064 | 11.89 |  |
| *drm1 drm2* | 21450 | 6907 | 11676 | 1083 | 12.98 |  |
| *drm1 drm2* | 9346 | 3108 | 4979 | 447 | 11.96 |  |
| *drm1 drm2* | 26098 | 8068 | 14732 | 1201 | 12.36 |  |
| *drm1 drm2* | 12050 | 3699 | 6775 | 592 | 13.19 |  |
| *drm1 drm2* | 19606 | 7093 | 9964 | 1106 | 13.22 |  |
| *drm1 drm2* | 10934 | 3682 | 5837 | 500 | 11.39 |  |
| *drm1 drm2* | 14487 | 4816 | 7725 | 752 | 12.99 |  |
| *drm1 drm2* | 14548 | 5083 | 7475 | 776 | 12.77 |  |
| *drm1 drm2* | 9530 | 3319 | 4752 | 563 | 13.91 | 5.37×10^-25^ |
| *kyp* | 11735 | 3959 | 6000 | 659 | 13.60 |  |
| *kyp* | 8641 | 3194 | 4229 | 454 | 11.94 |  |
| *kyp* | 12985 | 4503 | 6662 | 729 | 13.47 |  |
| *kyp* | 9370 | 3013 | 5041 | 486 | 13.24 |  |
| *kyp* | 14810 | 5220 | 7509 | 868 | 13.86 |  |
| *kyp* | 11232 | 4130 | 5321 | 764 | 15.22 |  |
| *kyp* | 14425 | 5082 | 7231 | 885 | 14.42 |  |
| *kyp* | 20123 | 7630 | 9475 | 1320 | 14.44 |  |
| *kyp* | 11248 | 3894 | 5850 | 611 | 13.15 |  |
| *kyp* | 15136 | 5309 | 7913 | 850 | 13.57 | 8.19×10^-60^ |
| *cmt3* | 15701 | 5196 | 8201 | 1018 | 16.04 |  |
| *cmt3* | 13957 | 5039 | 6775 | 930 | 15.22 |  |
| *cmt3* | 8091 | 2705 | 4123 | 555 | 16.63 |  |
| *cmt3* | 9077 | 3311 | 4352 | 610 | 15.18 |  |
| *cmt3* | 9281 | 3239 | 4370 | 546 | 13.40 |  |
| *cmt3* | 15695 | 4820 | 8409 | 904 | 14.93 |  |
| *cmt3* | 7323 | 2295 | 4008 | 408 | 14.55 |  |
| *cmt3* | 8060 | 2500 | 4372 | 490 | 15.84 |  |
| *cmt3* | 10156 | 3302 | 5564 | 587 | 14.87 |  |
| *cmt3* | 13760 | 4645 | 7046 | 933 | 16.43 |  |
| *cmt3* | 12086 | 4339 | 6089 | 701 | 13.56 |  |
| *cmt3* | 16911 | 6065 | 8648 | 1009 | 14.08 |  |
| *cmt3* | 16700 | 5875 | 8662 | 1016 | 14.61 |  |
| *cmt3* | 12962 | 4034 | 7282 | 757 | 15.59 | 3.00×10^-143^ |
| *drm1 drm2 cmt3* | 12433 | 4009 | 6454 | 811 | 16.24 |  |
| *drm1 drm2 cmt3* | 13963 | 4498 | 7233 | 904 | 16.10 |  |
| *drm1 drm2 cmt3* | 15658 | 2658 | 11401 | 686 | 19.84 |  |
| *drm1 drm2 cmt3* | 15401 | 5641 | 7424 | 996 | 14.63 |  |
| *drm1 drm2 cmt3* | 6534 | 2212 | 3355 | 473 | 17.55 |  |
| *drm1 drm2 cmt3* | 7845 | 2929 | 3722 | 510 | 14.46 |  |
| *drm1 drm2 cmt3* | 8803 | 3117 | 4338 | 620 | 16.35 |  |
| *drm1 drm2 cmt3* | 9279 | 3118 | 4693 | 605 | 15.71 |  |
| *drm1 drm2 cmt3* | 7918 | 2886 | 3896 | 500 | 14.48 | 1.40×10^-144^ |
| *kyp suvh5 suvh6* | 7685 | 2057 | 4759 | 408 | 16.38 |  |
| *kyp suvh5 suvh6* | 8649 | 2833 | 4544 | 611 | 17.61 |  |
| *kyp suvh5 suvh6* | 7506 | 1934 | 4682 | 394 | 16.56 |  |
| *kyp suvh5 suvh6* | 10735 | 3927 | 5052 | 791 | 16.46 |  |
| *kyp suvh5 suvh6* | 2981 | 1061 | 1471 | 214 | 16.65 |  |
| *kyp suvh5 suvh6* | 7571 | 2846 | 3570 | 527 | 15.39 |  |
| *kyp suvh5 suvh6* | 4145 | 1357 | 2193 | 263 | 15.90 |  |
| *kyp suvh5 suvh6* | 7653 | 2807 | 3679 | 504 | 14.87 |  |
| *kyp suvh5 suvh6* | 8711 | 3194 | 4168 | 645 | 16.67 |  |
| *kyp suvh5 suvh6* | 9724 | 3342 | 4837 | 683 | 16.60 |  |
| *kyp suvh5 suvh6* | 9243 | 3063 | 4730 | 714 | 18.85 |  |
| *kyp suvh5 suvh6* | 5859 | 2172 | 2725 | 430 | 16.21 |  |
| *kyp suvh5 suvh6* | 14135 | 5049 | 6868 | 1076 | 17.47 |  |
| *kyp suvh5 suvh6* | 6667 | 2315 | 3313 | 463 | 16.33 |  |
| *kyp suvh5 suvh6* | 8377 | 2641 | 4469 | 600 | 18.32 |  |
| *kyp suvh5 suvh6* | 6908 | 1999 | 4087 | 392 | 16.27 |  |
| *kyp suvh5 suvh6* | 8997 | 3026 | 4433 | 698 | 18.39 | 3.49×10^-248^ |
| *DDM1/ddm1* | 11043 | 3860 | 6646 | 295 | 7.15 |  |
| *DDM1/ddm1* | 9903 | 3548 | 5871 | 247 | 6.52 |  |
| *DDM1/ddm1* | 8691 | 2946 | 5412 | 152 | 4.88 |  |
| *DDM1/ddm1* | 3991 | 1161 | 2609 | 82 | 6.45 | 5.53×10^-72^ |
| *MET1/met1* | (Yelina et al. 2015) | (Yelina et al. 2015) | (Yelina et al. 2015) | (Yelina et al. 2015) | 8.60 |  |
| *MET1/met1* | (Yelina et al. 2015) | (Yelina et al. 2015) | (Yelina et al. 2015) | (Yelina et al. 2015) | 8.20 |  |
| *MET1/met1* | (Yelina et al. 2015) | (Yelina et al. 2015) | (Yelina et al. 2015) | (Yelina et al. 2015) | 8.10 |  |
| *MET1/met1* | (Yelina et al. 2015) | (Yelina et al. 2015) | (Yelina et al. 2015) | (Yelina et al. 2015) | 5.70 |  |
| *MET1/met1* | (Yelina et al. 2015) | (Yelina et al. 2015) | (Yelina et al. 2015) | (Yelina et al. 2015) | 5.70 |  |
| *MET1/met1* | (Yelina et al. 2015) | (Yelina et al. 2015) | (Yelina et al. 2015) | (Yelina et al. 2015) | 5.40 |  |
| *MET1/met1* | (Yelina et al. 2015) | (Yelina et al. 2015) | (Yelina et al. 2015) | (Yelina et al. 2015) | 5.20 |  |
| *MET1/met1* | (Yelina et al. 2015) | (Yelina et al. 2015) | (Yelina et al. 2015) | (Yelina et al. 2015) | 4.60 |  |
| *MET1/met1* | (Yelina et al. 2015) | (Yelina et al. 2015) | (Yelina et al. 2015) | (Yelina et al. 2015) | 4.50 |  |
| *MET1/met1* | (Yelina et al. 2015) | (Yelina et al. 2015) | (Yelina et al. 2015) | (Yelina et al. 2015) | 4.40 |  |
| *MET1/met1* | (Yelina et al. 2015) | (Yelina et al. 2015) | (Yelina et al. 2015) | (Yelina et al. 2015) | 4.30 |  |
| *MET1/met1* | (Yelina et al. 2015) | (Yelina et al. 2015) | (Yelina et al. 2015) | (Yelina et al. 2015) | 4.10 |  |
| *MET1/met1* | (Yelina et al. 2015) | (Yelina et al. 2015) | (Yelina et al. 2015) | (Yelina et al. 2015) | 4.00 |  |
| *MET1/met1* | (Yelina et al. 2015) | (Yelina et al. 2015) | (Yelina et al. 2015) | (Yelina et al. 2015) | 3.80 |  |
| *MET1/met1* | (Yelina et al. 2015) | (Yelina et al. 2015) | (Yelina et al. 2015) | (Yelina et al. 2015) | 3.70 |  |
| *MET1/met1* | (Yelina et al. 2015) | (Yelina et al. 2015) | (Yelina et al. 2015) | (Yelina et al. 2015) | 3.10 |  |
| *MET1/met1* | (Yelina et al. 2015) | (Yelina et al. 2015) | (Yelina et al. 2015) | (Yelina et al. 2015) | 2.50 |  |
| *MET1/met1* | (Yelina et al. 2015) | (Yelina et al. 2015) | (Yelina et al. 2015) | (Yelina et al. 2015) | 2.00 |  |
| *MET1/met1* | (Yelina et al. 2015) | (Yelina et al. 2015) | (Yelina et al. 2015) | (Yelina et al. 2015) | 1.90 |  |
| *MET1/met1* | (Yelina et al. 2015) | (Yelina et al. 2015) | (Yelina et al. 2015) | (Yelina et al. 2015) | 2.20 | nd |
| *met1* | (Yelina et al. 2015) | (Yelina et al. 2015) | (Yelina et al. 2015) | (Yelina et al. 2015) | 2.40 |  |
| *met1* | (Yelina et al. 2015) | (Yelina et al. 2015) | (Yelina et al. 2015) | (Yelina et al. 2015) | 2.10 |  |
| *met1* | (Yelina et al. 2015) | (Yelina et al. 2015) | (Yelina et al. 2015) | (Yelina et al. 2015) | 2.10 |  |
| *met1* | (Yelina et al. 2015) | (Yelina et al. 2015) | (Yelina et al. 2015) | (Yelina et al. 2015) | 1.30 | nd |

**Supplemental Table S2.** **CG, CHG and CHH DNA methylation levels in *CEN3* and correlation with cM in wild type and H3K9me2/non-CG DNA methylation mutants.** Mean cytosine methylation levels within *CEN3* were calculated from published data (Stroud et al. 2013). Pearson’s correlation coefficient was calculated between *CEN3* cM and levels of DNA methylation within *CEN3* in CG, CHG or CHH DNA sequence contexts. CHG showed a significant correlation (*P*=2.36×10^-3^), but CG and CHG did not show a significant correlation (n.s.).

| Genotype | *CEN3* CG methylation | *CEN3* CHG methylation | *CEN3* CHH methylation | *CEN3* cM |
| --- | --- | --- | --- | --- |
| Wild type (Col) | 0.71 | 0.37 | 0.10 | 11.17 |
| *cmt2* | 0.68 | 0.30 | 0.01 | 11.16 |
| *drm1 drm2* | 0.67 | 0.34 | 0.07 | 12.67 |
| *kyp* | 0.70 | 0.16 | 0.05 | 13.69 |
| *cmt3* | 0.69 | 0.04 | 0.06 | 15.07 |
| *drm1 drm2 cmt3* | 0.68 | 0.04 | 0.05 | 16.15 |
| *kyp suvh5 suvh6* | 0.66 | 0.05 | 0.03 | 16.76 |
| Correlation coefficient with *CEN3* cM | n.s. | -0.933 | n.s. |  |
|  |  |  |  |  |

**Supplemental Table S3.** **FTL interval *CTL5.11* count data and genetic distances for Col and *cmt3-11*.** Genetic distance is calculated as cM = 100 × (1 – (1 − 2(*N_G_*+*N_R_*)/*N_T_*)^1/2^). Where *N_G_* is the number of green alone seeds, *N_R_* is the number of red alone seeds and *N_T_* is the total number of seeds analysed. The number of recombinant (*N_G_*+*N_R_*) and non-recombinant (*N_RG_*+*N_--_*) counts for wild type and mutant genotypes were used to construct 2×2 contingency tables and *X^2^* tests performed to test for significant differences.

| Genotype | Green alone | Red alone | Both colours | No colour | Total | cM | *X2 P* |
| --- | --- | --- | --- | --- | --- | --- | --- |
| Col | 114 | 112 | 828 | 213 | 1,267 | 19.80 |  |
| Col | 182 | 173 | 1,317 | 340 | 2,012 | 19.56 |  |
| Col | 150 | 161 | 1,365 | 318 | 1,994 | 17.05 |  |
| Col | 174 | 191 | 1,345 | 330 | 2,040 | 19.87 |  |
| Col | 175 | 156 | 1,313 | 311 | 1,955 | 18.67 |  |
| Col | 181 | 182 | 1,398 | 336 | 2,097 | 19.14 | n.d. |
| *cmt3-11* | 101 | 105 | 721 | 151 | 1,078 | 21.40 |  |
| *cmt3-11* | 184 | 193 | 1,294 | 328 | 1,999 | 21.08 |  |
| *cmt3-11* | 178 | 192 | 1,302 | 352 | 2,024 | 20.35 |  |
| *cmt3-11* | 186 | 205 | 1,429 | 314 | 2,134 | 20.40 |  |
| *cmt3-11* | 136 | 167 | 1,003 | 221 | 1,527 | 22.34 |  |
| *cmt3-11* | 197 | 188 | 1,309 | 332 | 2,026 | 21.26 | 1.30×10^-3^ |

**Supplemental Table S4. FTL interval *LTL5.4* count data and genetic distances for Ler and *cmt3-7*.** Genetic distance is calculated as cM = 100 × (1 – (1 − 2(*N_G_*+*N_R_*)/*N_T_*)^1/2^). Where *N_G_* is the number of green alone seeds, *N_R_* is the number of red alone seeds and *N_T_* is the total number of seeds analysed. The number of recombinant (*N_G_*+*N_R_*) and non-recombinant (*N_RG_*+*N_--_*) counts for wild type and mutant genotypes were used to construct 2×2 contingency tables and *X^2^* tests performed to test for significant differences.

| Genotype | Green alone | Red alone | Both colours | No colour | Total | cM | *X^2^ P* |
| --- | --- | --- | --- | --- | --- | --- | --- |
| Ler | 93 | 80 | 1,441 | 431 | 2,045 | 8.85 |  |
| Ler | 104 | 88 | 1,531 | 472 | 2,195 | 9.17 |  |
| Ler | 108 | 94 | 1,465 | 428 | 2,095 | 10.16 |  |
| Ler | 109 | 104 | 1,297 | 355 | 1,865 | 12.16 |  |
| Ler | 121 | 108 | 1,445 | 516 | 2,190 | 11.07 | n.d. |
| *cmt3-7* | 125 | 114 | 1,456 | 435 | 2,130 | 11.93 |  |
| *cmt3-7* | 106 | 97 | 1,150 | 317 | 1,670 | 13.00 |  |
| *cmt3-7* | 153 | 133 | 1,390 | 353 | 2,029 | 15.26 |  |
| *cmt3-7* | 142 | 120 | 1,475 | 416 | 2,153 | 13.02 |  |
| *cmt3-7* | 132 | 133 | 1,494 | 432 | 2,191 | 12.93 | 2.09×10^-9^ |

**Supplemental Table S5. FTL interval *CEN3* count data and genetic distances for wild type and *cmt3* hybrid F_1_.** Genetic distance is calculated as cM = 100 × 2 × R_6_ / (R_2_ – (R_5_ – R_4_)). Where R_2_ is the total number of pollen analysed, R_4_ is the number of eYFP and RFP positive pollen, R_5_ is the number of eYFP and RFP negative pollen and R_6_ is the number of eYFP positive pollen. The number of recombinant (R_6_) and non-recombinant (R_4_) counts for wild type and mutant genotypes were used to construct 2×2 contingency tables and *X^2^* tests performed to test for significant differences. For comparison to inbred data see Supplemental Table S2.

| Genotype | Total (R_2_) | eYFP/RFP (R_4_) | No colour (R_5_) | eYFP (R_6_) | cM | *X^2^ P* |
| --- | --- | --- | --- | --- | --- | --- |
| Col/Ler F_1_ | 17,620 | 7,176 | 8,450 | 928 | 11.35 |  |
| Col/Ler F_1_ | 12,886 | 5,069 | 6,215 | 765 | 13.03 |  |
| Col/Ler F_1_ | 15,550 | 6,065 | 7,528 | 925 | 13.13 |  |
| Col/Ler F_1_ | 5,734 | 2,033 | 3,055 | 321 | 13.62 |  |
| Col/Ler F_1_ | 13,038 | 4,560 | 7,018 | 707 | 13.36 |  |
| Col/Ler F_1_ | 10,597 | 3,639 | 5,766 | 597 | 14.10 |  |
| Col/Ler F_1_ | 16,806 | 6,435 | 8,431 | 937 | 12.65 |  |
| Col/Ler F_1_ | 10,486 | 4,036 | 5,269 | 533 | 11.52 |  |
| Col/Ler F_1_ | 15,304 | 6,032 | 7,494 | 859 | 12.41 |  |
| Col/Ler F_1_ | 10,056 | 3,287 | 5,650 | 505 | 13.13 |  |
| Col/Ler F_1_ | 18,911 | 7,328 | 9,349 | 1,033 | 12.23 | n.d. |
| *cmt3-11*/*cmt3-7* F_1_ | 15,831 | 5,640 | 7,893 | 1,073 | 15.80 |  |
| *cmt3-11*/*cmt3-7* F_1_ | 14,877 | 5,082 | 7,715 | 1,012 | 16.53 |  |
| *cmt3-11*/*cmt3-7* F_1_ | 17,030 | 5,778 | 8,825 | 1,151 | 16.46 |  |
| *cmt3-11*/*cmt3-7* F_1_ | 21,453 | 8,044 | 10,139 | 1,595 | 16.48 |  |
| *cmt3-11*/*cmt3-7* F_1_ | 21,439 | 7,564 | 10,679 | 1,513 | 16.51 |  |
| *cmt3-11*/*cmt3-7* F_1_ | 17,185 | 6,566 | 7,928 | 1,300 | 16.43 |  |
| *cmt3-11*/*cmt3-7* F_1_ | 7,768 | 2,203 | 4,710 | 403 | 15.32 |  |
| *cmt3-11*/*cmt3-7* F_1_ | 6,136 | 1,748 | 3,634 | 349 | 16.42 |  |
| *cmt3-11*/*cmt3-7* F_1_ | 16,122 | 5,635 | 8,036 | 1,238 | 18.05 |  |
| *cmt3-11*/*cmt3-7* F_1_ | 15,313 | 5,090 | 8,112 | 1,004 | 16.34 | 1.27×10^-86^ |

**Supplemental Table S6. FTL interval *420* count data and genetic distances for wild type and *cmt3* inbred and hybrid F_1_ crosses.** Genetic distance is calculated as cM = 100 × (1 – (1 − 2(*N_G_*+*N_R_*)/*N_T_*)^1/2^). Where *N_G_* is the number of green alone seeds, *N_R_* is the number of red alone seeds and *N_T_* is the total number of seeds analysed. The number of recombinant (*N_G_*+*N_R_*) and non-recombinant (*N_RG_*+*N_--_*) counts for wild type and mutant genotypes were used to construct 2×2 contingency tables and *X^2^* tests performed to test for significant differences.

| Genotype | Green alone | Red alone | Both colours | No colour | Total | cM | *X^2^ P* |
| --- | --- | --- | --- | --- | --- | --- | --- |
| Wild type (Col) | 189 | 230 | 1,482 | 399 | 2,300 | 20.27 |  |
| Wild type (Col) | 263 | 254 | 1,824 | 423 | 2,764 | 20.89 |  |
| Wild type (Col) | 213 | 187 | 1,555 | 334 | 2,289 | 19.35 |  |
| Wild type (Col) | 173 | 196 | 1,417 | 357 | 2,143 | 19.03 |  |
| Wild type (Col) | 161 | 183 | 1,277 | 323 | 1,944 | 19.62 |  |
| Wild type (Col) | 151 | 148 | 1,236 | 277 | 1,812 | 18.15 |  |
| Wild type (Col) | 198 | 216 | 1,502 | 345 | 2,261 | 20.39 |  |
| Wild type (Col) | 205 | 230 | 1,384 | 319 | 2,138 | 22.99 |  |
| Wild type (Col) | 177 | 208 | 1,454 | 330 | 2,169 | 19.69 |  |
| Wild type (Col) | 223 | 256 | 1,499 | 349 | 2,327 | 23.3 |  |
| Wild type (Col) | 176 | 209 | 1,379 | 319 | 2,083 | 20.61 |  |
| Wild type (Col) | 188 | 213 | 1,458 | 376 | 2,235 | 19.93 |  |
| Wild type (Col) | 195 | 223 | 1,460 | 316 | 2,194 | 21.33 |  |
| Wild type (Col) | 198 | 208 | 1,339 | 325 | 2,070 | 22.04 |  |
| Wild type (Col) | 217 | 192 | 1,417 | 365 | 2,191 | 20.84 |  |
| Wild type (Col) | 225 | 251 | 1,536 | 372 | 2,384 | 22.5 |  |
| Wild type (Col) | 181 | 214 | 1,397 | 318 | 2,110 | 20.91 |  |
| Wild type (Col) | 200 | 231 | 1,498 | 352 | 2,281 | 21.13 |  |
| Wild type (Col) | 190 | 203 | 1,562 | 379 | 2,334 | 18.56 |  |
| Wild type (Col) | 217 | 214 | 1,545 | 381 | 2,357 | 20.36 |  |
| Wild type (Col) | 192 | 191 | 1,287 | 327 | 1,997 | 21.49 | n.d. |
| *cmt3* | 145 | 202 | 1,410 | 336 | 2,093 | 18.24 |  |
| *cmt3* | 180 | 207 | 1,523 | 348 | 2,258 | 18.93 |  |
| *cmt3* | 169 | 241 | 1,557 | 410 | 2,377 | 19.07 |  |
| *cmt3* | 183 | 234 | 1,525 | 394 | 2,336 | 19.81 |  |
| *cmt3* | 206 | 273 | 1,562 | 424 | 2,465 | 21.81 |  |
| *cmt3* | 233 | 245 | 1,582 | 366 | 2,426 | 22.16 |  |
| *cmt3* | 197 | 263 | 1,542 | 349 | 2,351 | 21.98 |  |
| *cmt3* | 214 | 199 | 1,529 | 423 | 2,365 | 19.33 |  |
| *cmt3* | 191 | 185 | 1,467 | 345 | 2,188 | 18.99 |  |
| *cmt3* | 213 | 177 | 1,425 | 343 | 2,158 | 20.09 |  |
| *cmt3* | 221 | 193 | 1,524 | 415 | 2,353 | 19.49 |  |
| *cmt3* | 194 | 209 | 1,605 | 400 | 2,408 | 18.44 | 0.651 |
| Col x Ler F_1_ | 133 | 121 | 1,507 | 476 | 2,237 | 12.08 |  |
| Col x Ler F_1_ | 129 | 131 | 1,479 | 424 | 2,163 | 12.85 |  |
| Col x Ler F_1_ | 123 | 126 | 1,495 | 384 | 2,128 | 12.48 |  |
| Col x Ler F_1_ | 148 | 150 | 1,562 | 466 | 2,326 | 13.76 |  |
| Col x Ler F_1_ | 138 | 113 | 1,438 | 408 | 2,097 | 12.79 |  |
| Col x Ler F_1_ | 127 | 128 | 1,591 | 439 | 2,285 | 11.86 | n.d. |
| *cmt3-11* x *cmt3-7* F_1_ | 120 | 114 | 1,619 | 496 | 2,349 | 10.51 |  |
| *cmt3-11* x *cmt3-7* F_1_ | 123 | 111 | 1,535 | 436 | 2,205 | 11.24 |  |
| *cmt3-11* x *cmt3-7* F_1_ | 126 | 113 | 1,465 | 403 | 2,107 | 12.07 |  |
| *cmt3-11* x *cmt3-7* F_1_ | 115 | 103 | 1,519 | 426 | 2,163 | 10.65 | 1.96×10^-3^ |

**Supplemental Table S7. SSLP genotyping pericentromeric intervals in Col x Ler F_2_ and *cmt3-11* × *cmt3-7* F_2_ populations.** Genotyping was performed by genotyping-by-sequencing (GBS) or manual genotyping of simple sequence length polymorphisms (SSLP).

| Chromosome 1 | 13.4 Mb - 16.2 Mb | |  | | |
| --- | --- | --- | --- | --- | --- |
| Genotype | Length (Mb) | Number of individuals | Crossovers | cM | cM/Mb |
| Col × Ler (G by S) | 2.8 | 437 | 16 | 3.66 | 1.31 |
| *cmt3-11* × *cmt3-7* (G by S) | 2.8 | 384 | 18 | 4.69 | 1.68 |
| Col × Ler (SSLP) | 2.8 | 727 | 36 | 4.95 | 1.77 |
| *cmt3-11* × *cmt3-7* (SSLP) | 2.8 | 737 | 55 | 7.46 | 2.67 |
|  |  |  |  |  |  |
| Chromosome 3 | 12.8 Mb - 17.1 Mb | |  | | |
| Genotype | Length (Mb) | Number of individuals | Crossovers | cM | cM/Mb |
| Col × Ler (G by S) | 4.3 | 437 | 79 | 18.08 | 4.20 |
| *cmt3-11* × *cmt3-7* (G by S) | 4.3 | 384 | 75 | 19.53 | 4.54 |
| Col × Ler (SSLP) | 4.3 | 711 | 140 | 19.69 | 4.58 |
| *cmt3-11* × *cmt3-7* (SSLP) | 4.3 | 725 | 157 | 21.66 | 5.04 |

**Supplemental Table S8. Total crossovers identified by genotyping-by-sequencing in wild type (Col/Ler) and *cmt3* (*cmt3-11/cmt3-7*) F_2_ populations.** Wild type crossovers (CO) were mapped by sequencing 245 F_2_ individuals to ~1-2× depth, which were combined with data from 192 F_2_ individuals (Choi et al. 2016). 384 *cmt3-11/cmt3-7* F_2_ individuals were sequenced to ~1-2× depth.

|  | Col/Ler  (n=437) | | *cmt3-11*/*cmt3-7*  (n=384) | |
| --- | --- | --- | --- | --- |
|  | CO/F_2_ | Total CO | CO/F_2_ | Total CO |
| Total | 7.59 | 3,320 | 7.23 | 2,803 |
| Chr1 | 1.84 | 802 | 1.76 | 677 |
| Chr2 | 1.29 | 562 | 1.21 | 466 |
| Chr3 | 1.46 | 639 | 1.44 | 554 |
| Chr4 | 1.25 | 547 | 1.28 | 493 |
| Chr5 | 1.76 | 770 | 1.60 | 613 |

**Supplemental Table S9. *Arabidopsis* centromeric, pericentromeric heterochromatin and euchromatic arm regions.** We define the centromeres genetically as contiguous regions flanking the TAIR10 centromeric assembly gaps that show an absence of crossovers in wild type (Copenhaver et al. 1999; Giraut et al. 2011; Salomé et al. 2012). We define the pericentromeric regions as regions flanking the centromeres with higher than chromosome average DNA methylation. The euchromatic arms constitute the remainder of the chromosomes, from the telomeres to the pericentromeres.

| Chr | North  Arm | North  Pericentromere | Centromere | South Pericentromere | South  Arm |
| --- | --- | --- | --- | --- | --- |
| 1 | 1 - 11,420,000 | 11,420,001 -  13,920,000 | 13,920,001-  15,970,000 | 15,970,001 -18,270,000 | 18,270,001 – 30,427,671 |
| 2 | 1 – 910,000 | 910,001 – 2,950,000 | 2,950,001 -  4,750,000 | 4,750,001 -  7,320,000 | 7,320,001 – 19,698,289 |
| 3 | 1 – 10,390,000 | 10,390,001 – 12,680,000 | 12,680,001 – 14,750,000 | 14,750,001 -  16,730,000 | 16,730,001 –  23,459,830 |
| 4 | 1 –  1,070,000 | 1,070,001 – 3,390,000 | 3,390,001 – 4,820,000 | 4,820,001 – 6,630,000 | 6,630,001 – 18,585,056 |
| 5 | 1 – 8,890,000 | 8,890,001 – 10,950,000 | 10,950,001 – 13,240,000 | 13,240,001 – 15,550,000 | 15,550,001 –  26,975,502 |

**Supplemental Table S10. Crossover numbers in chromosome arm, pericentromere or centromere regions identified in wild type or *cmt3* F_2_ mapping populations.** To test for significant differences in crossovers the counts within and outside a given region, e.g. arms, from wild type and *cmt3* were used to construct 2×2 contingency tables and *X^2^* tests performed.

| Wild type (*n*=437) | Chr1 | Chr2 | Chr3 | Chr4 | Chr5 | Total | *X^2^ P* |
| --- | --- | --- | --- | --- | --- | --- | --- |
| Arms | 621 | 382 | 496 | 415 | 588 | 2,502 |  |
| Pericentromeres | 181 | 180 | 143 | 132 | 182 | 818 |  |
| Centromeres | 0 | 0 | 0 | 0 | 0 | 0 |  |
| Total | 802 | 562 | 639 | 547 | 770 | 3,320 |  |
|  |  |  |  |  |  |  |  |
| *cmt3* (*n*=384) | Chr1 | Chr2 | Chr3 | Chr4 | Chr5 | Total |  |
| Arms | 511 | 316 | 387 | 361 | 436 | 2,011 | 1.50×10^-3^ |
| Pericentromeres | 163 | 149 | 166 | 125 | 176 | 779 | 5.60×10^-3^ |
| Centromeres | 3 | 1 | 1 | 7 | 1 | 13 | 2.63×10^-4^ |
| Total | 677 | 466 | 554 | 493 | 613 | 2,803 |  |

**Supplemental Table S11. Quantification of H3K9me2 immunostaining in leptotene stage meiotic cells of wild type and wild type, H3K9me2 and non-CG DNA methylation mutants.**

| Col | *cmt3-11* | *kyp suvh5 suvh6* |
| --- | --- | --- |
| 0.79 | 0.76 | 0 |
| 2.01 | 1.77 | 0 |
| 2.89 | 2.32 | 0 |
| 0.83 | 0.31 | 0 |
| 1.03 | 1.97 | 0 |
| 1.11 | 1.32 | 0 |
| 1.33 | 1.72 | 0 |
| 0.67 | 3.90 | 0 |
| 3.69 | 1.17 | 0 |
| 0.40 | 0.95 | 0 |
| 0.88 | 1.21 | 0 |
| 1.64 | 2.88 | 0 |
| 0.93 | 0.22 | 0 |
| 3.06 | 1.05 | 0 |
| 2.95 | 1.04 | 0 |
| 1.70 | 0.64 | 0 |
| 2.04 | 1.25 | 0 |
| 3.26 | 2.39 | 0 |
|  | 1.23 | 0 |
|  | 0.39 | 0 |
|  | 0.89 | 0 |

**Supplemental Table S12.** **Quantification of H3K9me2 immunostaining in somatic cells of wild type, H3K9me2/non-CG DNA methylation mutants**.

| Col | *cmt3* | *kyp suvh5 suvh6* |
| --- | --- | --- |
| 0.76 | 0.75 | 0 |
| 0.41 | 0.53 | 0 |
| 1.35 | 0.79 | 0 |
| 1.35 | 0.30 | 0 |
| 0.31 | 0.91 | 0 |
| 0.45 | 0.65 | 0 |
| 0.78 | 0.54 | 0 |
| 1.10 | 0.38 | 0 |
| 0.64 | 0.47 | 0 |
| 0.79 | 0.88 | 0 |
| 0.41 |  | 0 |
| 0.62 |  |  |

**Supplemental Table S13.** **Immunostained MLH1 foci counts at diakinesis in Col and *kyp suvh5 suvh6*.** MLH1 foci were classified as heterochromatic when they co-localized with the densely DAPI-staining chromocenters. A Mann-Whitney-Wilcoxon test was performed to test for significant differences in MLH1 foci number between wild type (Col) and *kyp suvh5 suvh6*.

| Cell | Wild type (Col) | | *kyp suvh5 suvh6* | |
| --- | --- | --- | --- | --- |
|  | Total MLH1 | Heterochromatic MLH1 | Total MLH1 | Heterochromatic MLH1 |
| 1 | 9 | 2 | 13 | 5 |
| 2 | 9 | 2 | 12 | 2 |
| 3 | 11 | 2 | 10 | 2 |
| 4 | 9 | 2 | 12 | 2 |
| 5 | 8 | 1 | 12 | 1 |
| 6 | 9 | 2 | 11 | 4 |
| 7 | 8 | 1 | 10 | 2 |
| 8 | 9 | 1 | 11 | 3 |
| 9 | 10 | 0 | 11 | 2 |
| 10 | 11 | 2 | 11 | 4 |
| 11 | 9 | 3 | 9 | 2 |
| 12 | 9 | 2 | 11 | 3 |
| 13 | 9 | 2 | 10 | 1 |
| 14 | 8 | 2 | 9 | 2 |
| 15 | 9 | 2 | 10 | 2 |
| 16 | 11 | 2 | 9 | 3 |
| 17 | 11 | 2 | 11 | 1 |
| 18 | 11 | 3 | 11 | 3 |
| 19 | 8 | 1 | 10 | 3 |
| 20 | 9 | 2 | 11 | 5 |
| 21 | 12 | 0 | 11 | 4 |
| 22 | 10 | 3 | 14 | 4 |
| 23 | 11 | 2 | 11 | 5 |
| 24 | 8 | 1 | 13 | 3 |
| 25 | 9 | 2 | 12 | 3 |
| 26 | 9 | 2 | 12 | 3 |
| 27 | 8 | 2 | 9 | 2 |
| 28 | 8 | 3 | 13 | 5 |
| 29 | 10 | 0 | 13 | 4 |
| 30 | 9 | 1 | 13 | 4 |
| 31 | 9 | 2 | 10 | 2 |
| 32 |  |  | 11 | 3 |
| 33 |  |  | 13 | 1 |
| 34 |  |  | 12 | 2 |
| 35 |  |  | 11 | 2 |
| 36 |  |  | 12 | 3 |
| 37 |  |  | 11 | 3 |
| 38 |  |  | 11 | 4 |
| 39 |  |  | 9 | 2 |
| 40 |  |  | 10 | 2 |
| 41 |  |  | 10 | 2 |
| 42 |  |  | 12 | 4 |
| 43 |  |  | 11 | 3 |
| 44 |  |  | 13 | 2 |
| 45 |  |  | 11 | 5 |
| 46 |  |  | 12 | 2 |
| 47 |  |  | 10 | 4 |
| 48 |  |  | 11 | 2 |
| Mean | 9.35 | 1.74 | 11.14 | 2.85 |
| St.Dev. | 1.14 | 1.25 | 0.81 | 1.15 |
| MWW *P* | n.d | 1.64×10^-7^ | n.d. | 4.22×10^-5^ |

**Supplemental Table S14.** **FTL interval *CEN3* count data and genetic distances for mutants in recombination and epigenetic pathways.** Genetic distance is calculated as cM = 100 x 2 x R_6_ / (R_2_ – (R_5_ – R_4_)). Where R_2_ is the total number of pollen analysed, R_4_ is the number of eYFP and RFP positive pollen, R_5_ is the number of eYFP and RFP negative pollen and R_6_ is the number of eYFP positive pollen. The number of recombinant (R_6_) and non-recombinant (R_4_) counts for wild type and mutant genotypes were used to construct 2×2 contingency tables and *X^2^* tests performed to test for significant differences.

| Genotype | Total (R_2_) | eYFP/RFP (R_4_) | No colour (R_5_) | eYFP (R_6_) | cM | *X^2^ P* |
| --- | --- | --- | --- | --- | --- | --- |
| Wild type (Col) | 7,019 | 1,977 | 4,183 | 284 | 11.80 |  |
| Wild type (Col) | 6,737 | 2,263 | 3,466 | 329 | 11.89 |  |
| Wild type (Col) | 5,355 | 1,675 | 3,003 | 218 | 10.83 |  |
| Wild type (Col) | 8,548 | 2,968 | 4,416 | 439 | 12.37 |  |
| Wild type (Col) | 8,602 | 2,817 | 4,752 | 367 | 11.01 |  |
| Wild type (Col) | 9,827 | 3,004 | 5,489 | 413 | 11.25 | n.d. |
| *cmt3* | 11,701 | 3,978 | 5,847 | 785 | 15.97 |  |
| *cmt3* | 8,634 | 2,718 | 4,526 | 553 | 16.20 |  |
| *cmt3* | 10,731 | 3,788 | 5,221 | 693 | 14.91 |  |
| *cmt3* | 11,008 | 3,961 | 5,224 | 808 | 16.58 |  |
| *cmt3* | 5,423 | 1,521 | 2,941 | 282 | 14.09 | 2.22×10^-28^ |
| *fancm* | 7,031 | 1,542 | 4,465 | 286 | 13.92 |  |
| *fancm* | 12,133 | 2,918 | 7,642 | 535 | 14.44 |  |
| *fancm* | 13,931 | 3,492 | 8,337 | 742 | 16.33 | 3.07 ×10^-21^ |
| *cmt3 fancm* | 14,306 | 3,345 | 8,674 | 805 | 17.93 |  |
| *cmt3 fancm* | 10,625 | 2,449 | 6,621 | 541 | 16.77 |  |
| *cmt3 fancm* | 12,988 | 3,454 | 7,328 | 848 | 18.61 | 1.65×10^-57^ |
| *zip4* | 6,873 | 2,026 | 4,031 | 25 | 1.03 |  |
| *zip4* | 5,553 | 1,646 | 3,296 | 23 | 1.18 |  |
| *zip4* | 4,665 | 1,390 | 2,754 | 17 | 1.03 | 2.30×10^-119^ |
| *cmt3 zip4* | 10,698 | 3,453 | 5,283 | 76 | 1.71 |  |
| *cmt3 zip4* | 2,767 | 484 | 1,231 | 31 | 3.07 |  |
| *cmt3 zip4* | 2,495 | 373 | 1,656 | 10 | 1.65 |  |
| *cmt3 zip4* | 2,392 | 260 | 1,630 | 14 | 2.74 |  |
| *cmt3 zip4* | 2,108 | 457 | 1,361 | 23 | 3.82 | 1.78×10^-83^ |
| *fancm zip4* | 9,706 | 3,234 | 5,452 | 354 | 9.46 |  |
| *fancm zip4* | 13,192 | 4,329 | 7,468 | 412 | 8.20 |  |
| *fancm zip4* | 14,638 | 4,481 | 8,711 | 428 | 8.22 | 6.29×10^-19^ |

**Supplemental Table S15.** **Seed set per silique for mutants in recombination and epigenetic pathways*.***

| Genotype | Silique 1 | Silique 2 | Silique 3 | Silique 4 | Silique 5 | Silique 6 | Silique 7 | Silique 8 | Plant average | Genotype Average |
| --- | --- | --- | --- | --- | --- | --- | --- | --- | --- | --- |
| Wild type (Col) | 47 | 43 | 42 |  |  |  |  |  | 44 |  |
| Wild type (Col) | 56 | 52 | 54 |  |  |  |  |  | 54 |  |
| Wild type (Col) | 48 | 46 | 45 |  |  |  |  |  | 46 | 48.11 |
| *zip4* | 5 | 1 | 2 | 2 | 3 | 3 | 1 | 4 | 3 |  |
| *zip4* | 5 | 2 | 3 | 3 | 5 | 2 | 2 | 1 | 3 |  |
| *zip4* | 3 | 4 | 3 | 2 | 4 | 3 | 2 | 4 | 3 | 2.88 |
| *cmt3* | 46 | 44 | 52 |  |  |  |  |  | 47 |  |
| *cmt3* | 54 | 42 | 45 |  |  |  |  |  | 47 |  |
| *cmt3* | 42 | 40 | 49 |  |  |  |  |  | 44 | 46.00 |
| *cmt3 zip4* | 5 | 5 | 4 | 6 | 5 | 6 | 5 | 1 | 4 |  |
| *cmt3 zip4* | 0 | 1 | 3 | 4 | 0 | 0 | 1 | 2 | 1 |  |
| *cmt3 zip4* | 1 | 0 | 1 | 2 | 2 | 1 | 4 | 3 | 2 | 2.58 |

**Supplemental Table S16. Mapping and analysis of SPO11-1-oligonucleotide reads.** The genotype and barcode for each library are listed, followed by (i) total sequenced reads (Total reads), (ii) trimmed reads following FastX processing (Trimmed reads), (iii) mapped reads from Bowtie2 (Mapped reads), (iv) alignments filtered for 2 or less mismatches (Mismatch filtered), (v) uniquely aligning reads (Uniquely aligning), (vi) uniquely aligning reads following deduplication (Unique rmdup). The lower part of the table lists the same libraries followed by (i) the number of multiply aligning reads (Multiply aligning), (ii) filtered multiply aligning reads selecting single alignments with MAPQ scores greater than 10 (Multiple unique fq10), (iii) the multiple-unique reads were then deduplicated (Multiple unique fq10 rmdup) and (iv) the final number of reads selected for analysis (Unique both rmdup). Finally, the adapter sequence and read length associated with each library are listed. For further detail please see accompanying manuscript (Choi et al, submitted), which describes the wild type Col libraries in more detail.

| Genotype | Library  barcode | Total  reads | Trimmed  reads | Mapped  reads | Mismatch  filtered | Uniquely aligning | Unique  rmdup |
| --- | --- | --- | --- | --- | --- | --- | --- |
| Col | RPI1 | 209,124,715 | 140,685,106 | 85,708,657 | 76,147,821 | 56,093,177 | 26,049,437 |
| Col | RPI3 | 205,166,702 | 172,856,279 | 131,601,991 | 123,150,472 | 89,791,279 | 12,620,592 |
| Col | RPI8 | 72,517,675 | 60,579,240 | 54,115,435 | 51,619,430 | 39,985,012 | 10,172,066 |
| *suvh456* | RPI34 | 81,135,212 | 19,474,163 | 15,584,160 | 15,031,140 | 11,008,046 | 4,563,437 |
| *suvh456* | RPI35 | 82,305,845 | 45,667,612 | 32,525,469 | 28,973,351 | 19,303,350 | 7,281,213 |
| Genotype | Library  barcode | Multiply  aligning | Multiple  unique fq10 | Multiple  unique fq10 rmdup | Unique  both rmdup | Adapter | Read Lengths |
| Col | RPI1 | 20,054,644 | 4,574,715 | 1,966,294 | 28,015,731 | GGGTGGAATTCTCGGGTGCCAAGGCT | 10-46 |
| Col | RPI3 | 33,359,193 | 7,258,667 | 889,638 | 13,510,230 | GGGTGGAATTCTCGGGTGCCAAGGCT | 10-46 |
| Col | RPI8 | 11,634,418 | 3,581,199 | 855,527 | 11,027,593 | GGGGTGGAATTCTCGGGTGCCAAGGCT | 10-69 |
| *suvh456* | RPI34 | 4,023,094 | 1,313,645 | 400,528 | 4,963,965 | GGGGTGGAATTCTCGGGTGCCAAGGCT | 10-69 |
| *suvh456* | RPI35 | 9,670,001 | 2,047,011 | 632,837 | 7,914,050 | GGGGTGGAATTCTCGGGTGCCAAGGCT | 11-69 |

**Supplemental Table S17. Correlation between SPO11-1-oligonucleotide libraries at varying physical scales**. Library size normalized SPO11-1-oligo coverage values were calculated in adjacent windows of the indicated physical size and correlated (Pearson’s) between libraries. All correlations were significant and the correlation coefficient is reported.

| Library 1 | RPI1 | RPI1 | RPI3 | RPI34 |
| --- | --- | --- | --- | --- |
| Library 2 | RPI3 | RPI8 | RPI8 | RPI35 |
| Genotype | Col | Col | Col | *kyp suvh5 suvh6* |
| 2 kb | 0.966 | 0.969 | 0.954 | 0.911 |
| 5 kb | 0.966 | 0.968 | 0.954 | 0.928 |
| 10 kb | 0.976 | 0.977 | 0.968 | 0.944 |
| 20 kb | 0.985 | 0.985 | 0.982 | 0.968 |
| 50 kb | 0.992 | 0.992 | 0.992 | 0.973 |
| 100 kb | 0.996 | 0.996 | 0.995 | 0.980 |

**Supplemental Table S18. SSLP Col/Ler deletion co-ordinates.**

| Marker Coordinates (Mb) | Left coordinate | Right coordinate | Deletion size (bp) |
| --- | --- | --- | --- |
| Chr1 (13.4) | 13,462,875 | 13,462,910 | 36 |
| Chr1 (16.2) | 16,263,108 | 16,263,141 | 34 |
| Chr3 (12.8) | 12,774,239 | 12,774,271 | 33 |
| Chr3 (17.1) | 17,088,210 | 17,088,409 | 200 |

**Supplemental Table S19. Oligonucleotides.**

| Chr | Primer name | Primer sequence | Col Product | Ler Product | Nearest Gene | Position (Mb) |
| --- | --- | --- | --- | --- | --- | --- |
| 1 | C4CEN-F | GCATAGGTTTCTACGTAAGAC | 148 | 112 | between AT1G36060 and AT1G36070 | 13.4 |
| 1 | C4CEN-R | TTCTATAGTAACGTTGACGC | | |  |  |
| 1 | 202-Chr1_16.2 F | CAAAGTTGTGTGAACCACTCCT | 160 | 126 | AT1G43160 | 16.2 |
| 1 | 203-Chr1_16.2 R | TCAATCCACAAGTGTTGAAAGATT | | |  |  |
| 3 | Chr3_12.8F | AATTGGGCCAGCTTTGTTTC | 242 | 209 | AT3G31403 | 12.8 |
| 3 | Chr3_12.8R | GGCGGATCCACATGTATTGA | | |  |  |
| 3 | 3-17088-F | GCTCTTGAGGTTTTAGGGTTGTT | 560 | 360 | AT3G46430 | 17.1 |
| 3 | 3-17088-R | TGCGTTCGCATGATTCAAAA | | |  |  |
